# Type-, dose-, and subgroup-specific associations of dairy intake with depressive and anxiety symptoms among middle school students

**DOI:** 10.3389/fnut.2026.1954062

**Published:** 2026-10-13

**Authors:** Meiqi Wang, Lianlong Yu, Zhaolu Liu, Shoujuan Zheng, Cuixia Lv, Jie Zhang, Afei Qin, Zhenchuang Tang, Suyun Li

**Affiliations:** 1 Department of Social Medicine and Health Management, School of Public Health, Cheeloo College of Medicine, Shandong University, Jinan, Shandong, China; 2 Shandong Center for Disease Control and Prevention, Jinan, China; 3 School of Public Health, Cheeloo College of Medicine, Shandong University, Jinan, Shandong, China; 4 School of Public Health, Shandong First Medical University & Shandong Academy of Medical Sciences, Jinan, China; 5 School of Public Health, Shandong Second Medical University, Weifang, China; 6 Department of Sociology, SUNY Buffalo State University, Buffalo, NY, United States; 7 Institute of Food and Nutrition Development, Ministry of Agriculture and Rural Affairs, Beijing, China

**Keywords:** adolescents, anxiety symptoms, dairy intake, depressive symptoms, middle school students, milk, yogurt

## Abstract

**Background:**

Depression and anxiety are major public health concerns among adolescents. Dairy products are important components of adolescent diets, but evidence on the associations of different dairy intake patterns with mental health symptoms remains limited. This study examined the associations of six dairy intake variables with depressive and anxiety symptoms among middle school students.

**Methods:**

This cross-sectional study included 135,138 middle school students in China. Dairy intake was assessed using six variables: frequencies of milk, yogurt, milk powder, and cheese/cheese cube intake; daily amount of milk, yogurt, and milk powder; and daily amount of cheese/cheese cubes. Depressive and anxiety symptoms were assessed using the Center for Epidemiologic Studies Depression scale and the Generalized Anxiety Disorder scale, respectively. Multivariable linear regression models, restricted cubic spline analyses, and interaction analyses by gender and urban-suburban context were conducted. The secondary analysis was approved by the Medical Ethics Committee of the Shandong Center for Disease Control and Prevention (Approval No. SDJK(K)2025-057-01).

**Results:**

Daily milk intake was reported by 27.5% of students, whereas daily yogurt, milk powder, and cheese/cheese cube intake were less common. Higher frequencies of milk and yogurt intake were associated with lower depressive and anxiety symptom scores. Compared with students who never consumed milk, daily milk consumers had lower depressive symptoms (β = −2.30, 95% CI:−2.50 to −2.10) and anxiety symptoms (β = −0.90, 95% CI:−0.99 to −0.81). The associations for milk powder, cheese/cheese cubes, and total dairy amount were more complex. Restricted cubic spline analyses suggested nonlinear associations, and interaction analyses indicated that several associations differed by gender and urban–suburban context.

**Conclusions:**

Dairy intake was differentially associated with depressive and anxiety symptoms among middle school students. More frequent milk and yogurt intake showed relatively consistent inverse associations with symptom scores, whereas associations for milk powder, cheese/cheese cubes, and amount-based dairy measures were less consistent and, for some measures, sensitive to model specification. These findings should be interpreted as observational associations and warrant further investigation in longitudinal studies with more detailed dietary assessment.

## 1 Introduction

Depression and anxiety are among the leading causes of illness and disability in adolescents and have become major global public health concerns (1). The prevalence of clinically elevated depressive and anxiety symptoms among children and adolescents was estimated to be 25.2% and 20.5%, respectively (2). Adolescence is a critical developmental period characterized by rapid changes in psychological functioning, neuroendocrine regulation, social relationships, and health-related behaviors, approximately half of all mental health problems in adulthood begin before the age of 18 years (1, 3). During adolescence, individuals may be particularly vulnerable to multiple influences, including academic pressure, family environment, peer relationships, insufficient sleep, physical inactivity, and unhealthy dietary behaviors. Depressive and anxiety symptoms not only cause immediate emotional distress but may also impair academic performance, interpersonal relationships, and the formation of healthy behaviors, predict poorer mental health outcomes in adulthood, and increase the risk of self-harm and suicidal behaviors (4–8). Therefore, identifying modifiable lifestyle-related factors associated with adolescent mental health is important for school-based health promotion and early prevention.

Dietary behavior is an increasingly recognized modifiable factor in research on child and adolescent mental health. Previous studies have shown that higher diet quality, greater fruit and vegetable intake, and lower consumption of sugar-sweetened beverages and ultra-processed foods are generally associated with lower levels of depressive or anxiety symptoms (9–12). Dairy products are important components of adolescents' diets and typically include milk, yogurt, milk powder, cheese, and related products. Dairy products are rich in high-quality protein, calcium, B vitamins, vitamin D, phosphorus, potassium, and, in some products, fermentation-derived bioactive compounds and probiotics. These nutrients and bioactive components may influence emotional regulation through pathways involving neurotransmitter synthesis, inflammation, oxidative stress, the gut microbiota, and the gut-brain axis (13–15). Therefore, dairy intake may be relevant not only to skeletal growth and physical development but also to mental health status among adolescents.

However, evidence on the association between dairy intake and depressive or anxiety symptoms remains limited and inconsistent. Some studies have reported that higher dairy or milk intake is associated with lower levels of depressive and anxiety symptoms (16, 17). Other studies suggest that associations may differ across dairy types, with fermented dairy products, regular milk, cheese, and total dairy intake potentially showing distinct associations with mental health outcomes (18–21). In addition, existing studies have mainly focused on adults or college students (20, 22, 23) whereas large-scale evidence among middle school students remains relatively limited. Moreover, it remains unclear whether different patterns exist between dairy intake frequency and amount in relation to depressive and anxiety symptoms, whether nonlinear dose-response associations are present, and whether these associations vary by gender or urban-suburban context.

To address these gaps, the present study used data from a large-scale cross-sectional survey of middle school students conducted in 2024 to systematically examine the associations of six dairy-related variables with depressive and anxiety symptoms. Given the observational and cross-sectional design, the primary objective of this study was associative rather than causal or predictive; accordingly, all estimates were interpreted as cross-sectional associations. Specifically, this study aimed to: (1) describe the frequency and amount of dairy intake among middle school students; (2) evaluate the associations between different dairy intake variables and depressive and anxiety symptom scores; (3) evaluate overall trends and explore potential nonlinear patterns in these associations; and (4) further examine whether the associations varied by gender and urban-suburban context. This study may provide large-sample evidence for understanding the relationship between dairy intake and adolescent mental health and provide evidence to inform future research on adolescent dietary patterns and mental health.

## 2 Materials and methods

### 2.1 Study design and population

This cross-sectional study was based on data from the 2024 Surveillance of Students' Common Diseases and Health Influencing Factors (SSCDHIF) conducted in Shandong Province, China. The surveillance program is an annual school-based survey designed to monitor common health problems and related behavioral factors among children and adolescents, thereby providing evidence for student health promotion and disease prevention (5). A multistage stratified cluster sampling strategy was used. Standardized paper-based questionnaires were administered by uniformly trained investigators. Completed questionnaires were collected by teachers and reviewed by investigators, and questionnaires with incomplete or inconsistent responses were checked and corrected when possible.

The present analysis focused on middle school students, including students from junior high schools, senior high schools, and vocational high schools. In 2024, a total of 156,281 middle school students participated in this survey. Within this surveillance program, dairy intake and parental education information were collected through a separate questionnaire module from the main student health survey. Among the 156,281 participants who participated in the 2024 surveillance, 21,143 participants were excluded because they had missing information on all dairy intake items and both father's and mother's education levels, resulting in a final analytic sample of 135,138 participants from 491 middle schools. A comparison of demographic characteristics, health behaviors, dairy intake variables, and mental health outcomes between included and excluded participants is presented in Supplementary Table S1. Compared with included participants, excluded participants were slightly younger, more likely to be from junior high schools and suburban areas. The remaining missing values were imputed using the mode, including those for history of smoking (*n* = 13) and unhealthy diet (*n* = 6).

The 2024 surveillance was conducted as part of the national SSCDHIF program, which had been reviewed and approved by the National Expert Committee before implementation of the field survey. The present secondary analysis of the previously collected surveillance data was subsequently approved by the Medical Ethics Committee of the Shandong Center for Disease Control and Prevention (Approval No. SDJK(K)2025-057-01). Before participation in the 2024 surveillance, participants aged 18 years or older provided written informed consent. For participants younger than 18 years, written informed consent was obtained from a parent or legal guardian, and assent was obtained from the student.

### 2.2 Measures

#### 2.2.1 Dairy intake

Dairy intake was assessed using six variables covering both frequency and amount of dairy consumption. The dairy-related questions were derived from the standardized questionnaire of the national SSCDHIF. Participants were asked about their dairy consumption during the past month. The exact questions and response options were as follows:

For frequency of consumption, participants were asked: “How often did you consume the following dairy products during the past month?” Four separate items assessed the frequency of milk, yogurt, milk powder, and cheese/cheese cube intake. The response options for each item were: never; less than once per month; 1–3 days/week; 4–6 days/week; and daily. For analysis, these responses were categorized into five levels: never, less than once per month, 1–3 days/week, 4–6 days/week, and daily.

For amount of consumption, participants were asked: “How much of the following dairy products did you consume per day?” The response options for milk, yogurt, and milk powder were never, 0.5 units/day, 1 unit/day, 1.5 units/day, 2 units/day, 2.5 units/day, and ≥3 units/day (with 1 unit corresponding to 200 ml). These responses were treated as a continuous variable and converted into 200 ml/day units for analysis. For cheese/cheese cubes, participants were asked about the daily amount consumed, with response options of never, 1–3 pieces, 4–6 pieces, 7–9 pieces, 10–12 pieces, 13–15 pieces, and >15 pieces. These categories were retained as categorical variables in the analyses.

#### 2.2.2 Depressive and anxiety symptoms

Depressive symptoms were assessed using the 20-item Center for Epidemiologic Studies Depression Scale (24). Items were scored from 0 to 3 after reverse coding of positively worded items, and the total score ranged from 0 to 60, with higher scores indicating more severe depressive symptoms. Anxiety symptoms were assessed using the 7-item Generalized Anxiety Disorder scale, with higher scores indicating more severe anxiety symptoms (25). Each item was scored from 0 to 3, and the total score ranged from 0 to 21, with higher scores indicating more severe anxiety symptoms. In line with established clinical thresholds, a CES-D score of ≥16 and a GAD-7 score of ≥10 were used to define elevated symptom levels. In the present sample, Cronbach's alpha coefficients were 0.877 for the CES-D and 0.937 for the GAD-7. Both depressive and anxiety symptoms were analyzed as continuous outcomes.

#### 2.2.3 Covariates

Covariates included demographic, health-related behavioral factors, and parental education. Demographic variables included age, gender, ethnicity, education level, region, and living on campus. Gender was coded as girls and boys, ethnicity as Han and other ethnicities, education level as junior high school and senior high school, and region as urban and suburban. Body mass index (BMI) was calculated using measured height and weight. Health-related behavioral factors included history of smoking, history of drinking, physical inactivity, low fruit or vegetable intake, and unhealthy diet. Parental education was assessed separately for fathers and mothers and categorized as junior high school or below and senior high school or above. Covariates were selected using a directed acyclic graph (DAG) based on hypothesized relationships among dairy intake, depressive and anxiety symptoms, and potential confounding factors. The resulting minimal sufficient adjustment set included all covariates listed above (Supplementary Figure S1).

### 2.3 Statistical analysis

Continuous variables were summarized as means and standard deviations, and categorical variables were summarized as frequencies and percentages. In univariate analyses, differences in depressive and anxiety symptom scores across categorical variables were examined using one-way analysis of variance. For continuous variables, Pearson correlation analysis was used to examine their associations with depressive and anxiety symptom scores.

All analyses were designed to characterize cross-sectional associations rather than to estimate causal effects or develop individual-level prediction models. Multivariable linear regression models were then used to examine the associations of dairy intake variables with depressive and anxiety symptom scores. Regression coefficients and 95% confidence intervals were reported. In the main model, the six dairy intake variables were included simultaneously. Variance inflation factors (VIFs) were calculated to assess multicollinearity, and all VIFs were below 5, indicating no substantial multicollinearity. To assess whether the associations were sensitive to simultaneous adjustment for other dairy variables, additional models were fitted by entering each dairy intake variable separately (Models D1-D6; Models A1-A6). All regression models adjusted for age, gender, ethnicity, education level, region, BMI, living on campus, history of smoking, history of drinking, physical inactivity, low fruit or vegetable intake, unhealthy diet, father's education level, and mother's education level. Model diagnostics for the primary linear regression models included R^2^, adjusted R^2^, root mean square error (RMSE), root mean square error (MAE), standardized residuals, Cook's distance, the Breusch-Pagan test for heteroscedasticity, and the Ramsey RESET test for potential functional-form misspecification.

Three sensitivity analyses were conducted to assess the robustness of the findings. First, multiple imputation (MI) was performed for variables with missing values, including six dairy intake variables and selected covariates with missing information. MI by chained equations (MICE) was conducted using five imputed datasets, with five iterations performed for each imputation. Ordinal dairy frequency variables were imputed using proportional odds logistic regression models, whereas binary variables were imputed using logistic regression models. All variables included in the regression models were incorporated into the imputation procedure to preserve the correlation structure among variables. Multivariable linear regression models identical to the main analyses were subsequently fitted within each imputed dataset, and estimates were pooled using Rubin's rules. Second, given the non-negative integer nature and overdispersion of the CES-D and GAD-7 scores, multivariable negative binomial regression models were fitted as an alternative specification of the continuous symptom outcomes, with results expressed as incidence rate ratios (IRRs) and 95% CIs. Third, depressive and anxiety symptoms were dichotomized according to validated cut-offs, and multivariable logistic regression models were fitted, with results expressed as odds ratios (ORs) and 95% CIs. All sensitivity models included the six dairy intake variables simultaneously and used the same covariate adjustment strategy as the primary analyses.

To evaluate monotonic trends across ordered dairy intake categories without assuming equal quantitative distances between adjacent categories, adjusted trend analyses were additionally performed. For the four dairy intake frequency variables and the categorical amount of cheese/cheese cubes, distribution-based ridit scores were assigned according to the observed cumulative distribution of the ordered categories. For the combined daily amount of milk, yogurt, and milk powder, the original quantitative values were modeled continuously. Each dairy exposure was entered separately into a multivariable linear regression model and adjusted for covariates. The P for trend was obtained from the coefficient of the corresponding ridit score or continuous quantitative exposure. For ordinal exposures, an overall *P* value was additionally obtained by modeling the exposure as a categorical variable. To account for multiple testing, Benjamini-Hochberg false discovery rate (FDR) correction was applied separately to the six trend tests for depressive symptoms and the six trend tests for anxiety symptoms. Restricted cubic spline (RCS) models were further used to explore potential nonlinear dose-response associations between dairy intake variables and depressive or anxiety symptom scores. For each exposure, models with 3, 4, and 5 knots were compared, and the model with the lowest Akaike information criterion (AIC) was selected. In the main RCS models, all six dairy variables and covariates were included simultaneously. In supplementary RCS models, each dairy variable was entered separately together with covariates.

Interaction analyses were considered exploratory and were conducted to examine whether the associations between dairy intake and depressive or anxiety symptoms differed by gender and region. In each interaction model, all six dairy intake variables and covariates were included simultaneously. For categorical dairy variables, the overall P for interaction was obtained from a joint test of the corresponding interaction terms, whereas for the continuous dairy amount variable, it was based on the product term. To account for multiple testing, FDR correction was applied separately to the six overall interaction tests within each outcome-modifier combination. FDR-adjusted *P* values were used to evaluate evidence of interaction. To facilitate interpretation of the interaction effects, subgroup-specific multivariable linear regression models were additionally fitted separately by gender and region.

To account for potential within-school clustering arising from the multistage cluster sampling design, additional sensitivity analyses were conducted using linear mixed-effects models with a random intercept for school. The models included the same six dairy intake variables and covariates. The gender and region interaction analyses were also repeated using school-level random-intercept models, with FDR correction applied to the overall interaction tests using the same approach.

All statistical tests were two-sided. For the exploratory interaction analyses, an FDR-adjusted *P* < 0.05 was used as the threshold for evidence of interaction. Given the large sample size, statistical significance alone was not considered evidence of substantive importance. Interpretation therefore emphasized the magnitude and direction of effect estimates and their 95% confidence intervals, with *P* values (*P* < 0.05) considered supplementary. Statistical analyses were performed using R software.

## 3 Results

### 3.1 Description of participants and univariate analyses

A total of 135,138 middle school students were included in the analysis. The mean age was 14.41 ± 1.87 years, and 70,708 students were boys (52.3%). Most participants were Han ethnicity (98.5%). Junior high school students accounted for 55.4% of the sample, and 50.3% of students were from urban areas. The mean BMI was 22.17 ± 4.75 kg/m^2^. Overall, 36.1% of students lived on campus, 2.9% reported a history of smoking, and 12.7% reported a history of drinking. The mean depressive symptom score was 9.83 ± 8.72, and the mean anxiety symptom score was 2.87 ± 3.96. Using the validated cut-offs, 23,978 participants (17.7%) had elevated depressive symptoms and 8,253 (6.1%) had elevated anxiety symptoms, respectively. Depressive and anxiety symptom scores were strongly correlated with each other (*r* = 0.733, *P* < 0.001). Participant characteristics and univariate associations with depressive and anxiety symptoms are shown in Table 1. Girls, senior high school students, students living on campus, students with a history of smoking or drinking, students with low fruit or vegetable intake, and students with unhealthy diet had higher depressive and anxiety symptom scores.

**Table 1:** Characteristics of participants and univariate associations with depressive and anxiety symptoms.

Variables	Total	Depressive symptoms			Anxiety symptoms		
		Mean ±**SD**	**F/r**	***P*** **value**	**Mean** ±**SD**	**F/r**	***P*** **value**
Number	135,138 (100.0)						
Age	14.41 ± 1.87		0.118	< 0.001		0.141	< 0.001
Gender			394.750	< 0.001		1,711.400	< 0.001
Boys	70,708 (52.3)	9.39 ± 8.31			2.44 ± 3.77		
Girls	64,430 (47.7)	10.33 ± 9.12			3.33 ± 4.10		
Ethnicity			0.100	0.756		0.580	0.447
Han	133,141 (98.5)	9.83 ± 8.72			2.87 ± 3.96		
Other	1,997 (1.5)	9.89 ± 8.63			2.93 ± 4.04		
Education level			1,432.960	< 0.001		2,333.650	< 0.001
Junior high school	74,827 (55.4)	9.03 ± 8.34			2.40 ± 3.75		
Senior high school	60,311 (44.6)	10.83 ± 9.06			3.44 ± 4.14		
Region			1.200	0.273		7.340	0.007
Urban	68,036 (50.3)	9.81 ± 8.78			2.90 ± 3.99		
Suburban	67,102 (49.7)	9.86 ± 8.65			2.84 ± 3.93		
BMI	22.17 ± 4.75		0.024	< 0.001		0.019	< 0.001
Living on campus			622.250	< 0.001		439.040	< 0.001
Yes	48,791 (36.1)	10.62 ± 8.77			3.17 ± 4.04		
No	86,347 (63.9)	9.39 ± 8.66			2.70 ± 3.90		
History of smoking			2,652.940	< 0.001		1,585.690	< 0.001
Yes	3,919 (2.9)	16.83 ± 11.69			5.34 ± 5.39		
No	131,219 (97.1)	9.63 ± 8.52			2.79 ± 3.89		
History of drinking			6,638.150	< 0.001		5,827.140	< 0.001
Yes	17,182 (12.7)	14.78 ± 10.89			4.98 ± 4.84		
No	117,956 (87.3)	9.11 ± 8.10			2.56 ± 3.72		
Physical inactivity			29.600	< 0.001		48.850	< 0.001
Yes	118,292 (87.5)	9.88 ± 8.66			2.90 ± 3.93		
No	16,846 (12.5)	9.49 ± 9.11			2.67 ± 4.13		
Low fruit or vegetable intake			4,351.060	< 0.001		1,753.020	< 0.001
Yes	51,689 (38.2)	11.79 ± 9.19			3.44 ± 4.30		
No	83,449 (61.8)	8.62 ± 8.18			2.51 ± 3.69		
Unhealthy diet			2,757.770	< 0.001		1,957.560	< 0.001
Yes	16,877 (12.5)	13.10 ± 10.16			4.12 ± 4.78		
No	118,261 (87.5)	9.37 ± 8.39			2.69 ± 3.80		
Father's education level			493.620	< 0.001		223.080	< 0.001
Junior high school or below	58,074 (43.0)	10.44 ± 8.78			3.05 ± 4.02		
Senior high school or above	77,064 (57.0)	9.38 ± 8.64			2.73 ± 3.91		
Mother's education level			614.440	< 0.001		364.960	< 0.001
Junior high school or below	64,908 (48.0)	10.44 ± 8.79			3.08 ± 4.03		
Senior high school or above	70,230 (52.0)	9.27 ± 8.60			2.67 ± 3.88		
Depressive symptoms	9.83 ± 8.72					0.733	< 0.001
Anxiety symptoms	2.87 ± 3.96		0.733	< 0.001			

BMI, body mass index; SD, standard deviation.

The distribution of dairy intake is shown in Table 2. Regarding dairy intake, 27.5% of students reported daily milk intake, while 7.3% reported never drinking milk. Yogurt intake was less frequent, with 10.5% reporting daily yogurt intake and 13.7% reporting never consuming yogurt. Most students reported never consuming milk powder (68.9%) or cheese/cheese cubes (63.5%). The mean daily amount of milk, yogurt, and milk powder was 1.17 ± 0.71 units of 200 ml. For cheese/cheese cubes, 65.8% of students reported no daily intake, while 21.7% reported consuming 1–3 pieces per day.

**Table 2:** Distribution of dairy intake variables by gender and region.

**Variables**	Total [*n* (%)/mean (SD)]	Gender		Region	
		Boys	Girls	Urban	Suburban
Frequency of milk					
Never	9,888 (7.3)	4,327 (6.1)	5,561 (8.6)	5,195 (7.6)	4,693 (7.0)
Less than once per month	14,846 (11.0)	6,660 (9.4)	8,186 (12.7)	7,713 (11.3)	7,133 (10.6)
1–3 days/week	46,203 (34.2)	22,246 (31.5)	23,957 (37.2)	23,601 (34.7)	22,602 (33.7)
4–6 days/week	27,080 (20.0)	14,869 (21.0)	12,211 (19.0)	13,456 (19.8)	13,624 (20.3)
Daily	37,121 (27.5)	22,606 (32.0)	14,515 (22.5)	18,071 (26.6)	19,050 (28.4)
Frequency of yogurt					
Never	18,457 (13.7)	11,765 (16.6)	6,692 (10.4)	8,820 (13.0)	9,637 (14.4)
Less than once per month	31,866 (23.6)	16,195 (22.9)	15,671 (24.3)	15,824 (23.3)	16,042 (23.9)
1–3 days/week	52,995 (39.2)	25,967 (36.7)	27,028 (41.9)	27,141 (39.9)	25,854 (38.5)
4–6 days/week	17,581 (13.0)	8,684 (12.3)	8,897 (13.8)	8,936 (13.1)	8,645 (12.9)
Daily	14,239 (10.5)	8,097 (11.5)	6,142 (9.5)	7,315 (10.8)	6,924 (10.3)
Frequency of milk powder					
Never	93,071 (68.9)	48,718 (68.9)	44,353 (68.8)	46,919 (69.0)	46,152 (68.8)
Less than once per month	13,793 (10.2)	6,474 (9.2)	7,319 (11.4)	6,669 (9.8)	7,124 (10.6)
1–3 days/week	15,198 (11.2)	7,844 (11.1)	7,354 (11.4)	7,718 (11.3)	7,480 (11.1)
4–6 days/week	5,931 (4.4)	3,297 (4.7)	2,634 (4.1)	3,185 (4.7)	2,746 (4.1)
Daily	7,145 (5.3)	4,375 (6.2)	2,770 (4.3)	3,545 (5.2)	3,600 (5.4)
Frequency of cheese/cheese cubes					
Never	85,842 (63.5)	46,251 (65.4)	39,591 (61.4)	42,284 (62.1)	43,558 (64.9)
Less than once per month	23,032 (17.0)	10,135 (14.3)	12,897 (20.0)	12,004 (17.6)	11,028 (16.4)
1–3 days/week	15,972 (11.8)	8,147 (11.5)	7,825 (12.1)	8,284 (12.2)	7,688 (11.5)
4–6 days/week	5,110 (3.8)	2,893 (4.1)	2,217 (3.4)	2,759 (4.1)	2,351 (3.5)
Daily	5,182 (3.8)	3,282 (4.6)	1,900 (2.9)	2,705 (4.0)	2,477 (3.7)
Daily amount of milk, yogurt, and milk powder (200ml)	1.17 (0.71)	1.20 (0.74)	1.14 (0.67)	1.17 (0.71)	1.17 (0.70)
Daily amount of cheese/cheese cubes					
Never	88,894 (65.8)	47,762 (67.5)	41,132 (63.8)	43,843 (64.4)	45,051 (67.1)
1–3 pieces	29,344 (21.7)	13,501 (19.1)	15,843 (24.6)	15,162 (22.3)	14,182 (21.1)
4–6 pieces	9,558 (7.1)	5,073 (7.2)	4,485 (7.0)	5,051 (7.4)	4,507 (6.7)
7–9 pieces	3,358 (2.5)	1,881 (2.7)	1,477 (2.3)	1,704 (2.5)	1,654 (2.5)
10–12 pieces	2,392 (1.8)	1,427 (2.0)	965 (1.5)	1,431 (2.1)	961 (1.4)
13–15 pieces	820 (0.6)	557 (0.8)	263 (0.4)	469 (0.7)	351 (0.5)
>15 pieces	772 (0.6)	507 (0.7)	265 (0.4)	376 (0.6)	396 (0.6)

SD, standard deviation.

The unadjusted distributions of depressive and anxiety symptom scores across dairy intake categories are presented in Supplementary Table S2. Milk intake showed the clearest descriptive pattern: mean depressive symptom scores decreased from 12.57 ± 10.46 among students who never consumed milk to 8.87 ± 8.25 among daily consumers, while mean anxiety symptom scores decreased from 3.97 ± 4.83 to 2.36 ± 3.76. Yogurt intake showed a broadly similar, although less monotonic, pattern. In contrast, descriptive patterns for milk powder and cheese/cheese cubes were more heterogeneous across intake categories. The correlations of the combined daily amount of milk, yogurt, and milk powder with depressive and anxiety symptom scores were very small (*r* = −0.034 and−0.006, respectively).

### 3.2 Multivariable regression analyses

The associations of dairy intake variables with depressive and anxiety symptom scores in the simultaneous multivariable linear regression models are shown in Table 3. All variance inflation factors were below 5, suggesting no substantial multicollinearity among the independent variables. In the primary mutually adjusted models, in which all six dairy variables and covariates were included simultaneously, milk intake frequency showed the largest and most consistent inverse estimates (Table 3). Compared with students who never consumed milk, daily milk intake corresponded to a 2.30-point lower depressive symptom score (β = −2.30, 95% CI:−2.50 to −2.10) and a 0.90-point lower anxiety symptom score (β = −0.90, 95% CI:−0.99 to −0.81). These estimates were similar to those from the single-exposure covariate-adjusted models (β = −2.31, 95% CI:−2.50 to −2.13 for depressive symptoms; β = −1.02, 95% CI:−1.10 to −0.93 for anxiety symptoms; Supplementary Tables S4–S5), indicating little change after mutual adjustment for other dairy variables. Yogurt intake frequency also showed inverse estimates, although of smaller magnitude. For daily yogurt intake, the estimates were β = −0.88 (95% CI:−1.09 to −0.68) for depressive symptoms and β = −0.35 (95% CI:−0.45 to −0.26) for anxiety symptoms in the mutually adjusted models. Corresponding estimates in the single-exposure models were−0.97 (95% CI:−1.15 to −0.78) and−0.84 (95% CI:−0.93 to −0.76), respectively, suggesting some attenuation, particularly for anxiety symptoms, after accounting for other dairy intake measures.

**Table 3:** Multivariable linear regression associations of dairy intake variables with depressive and anxiety symptoms in the primary mutually adjusted models.

Variables	Depressive symptoms		Anxiety symptoms	
	Beta (95% CI)	***P***	**Beta (95% CI)**	***P***
Age	0.28 (0.23, 0.32)	< 0.001	0.16 (0.14, 0.18)	< 0.001
Gender (Ref. = Girls)	−1.61 (−1.70,−1.52)	< 0.001	−1.10 (−1.14,−1.06)	< 0.001
Ethnicity (Ref. = Other)	−0.11 (−0.47, 0.25)	0.552	−0.07 (−0.23, 0.10)	0.435
Education level (Ref. = Junior high school)	−0.39 (−0.56,−0.22)	< 0.001	0.10 (0.03, 0.18)	0.009
Region (Ref. = Suburban)	−0.26 (−0.35,−0.17)	< 0.001	−0.10 (−0.14,−0.06)	< 0.001
BMI	0.03 (0.02, 0.04)	< 0.001	0.01 (0.01, 0.01)	< 0.001
Living on campus (Ref. = No)	−0.21 (−0.31,−0.10)	< 0.001	−0.18 (−0.23,−0.13)	< 0.001
History of smoking (Ref. = No)	3.54 (3.26, 3.81)	< 0.001	1.14 (1.01, 1.26)	< 0.001
History of drinking (Ref. = No)	4.43 (4.29, 4.57)	< 0.001	1.93 (1.87, 2.00)	< 0.001
Physical inactivity (Ref. = No)	0.32 (0.18, 0.45)	< 0.001	0.12 (0.06, 0.18)	< 0.001
Low fruit or vegetable intake (Ref. = No)	2.37 (2.28, 2.47)	< 0.001	0.63 (0.59, 0.67)	< 0.001
Unhealthy diet (Ref. = No)	2.78 (2.65, 2.92)	< 0.001	1.06 (1.00, 1.13)	< 0.001
Father's education level (Ref. = Junior high school or below)	−0.29 (−0.40,−0.17)	< 0.001	−0.03 (−0.08, 0.02)	0.237
Mother's education level (Ref. = Junior high school or below)	−0.40 (−0.51,−0.29)	< 0.001	−0.14 (−0.19,−0.09)	< 0.001
**Frequency of milk (Ref**. = **Never)**				
Less than once per month	−0.52 (−0.74,−0.31)	< 0.001	−0.20 (−0.30,−0.10)	< 0.001
1–3 days/week	−1.80 (−1.99,−1.61)	< 0.001	−0.72 (−0.80,−0.63)	< 0.001
4–6 days/week	−1.92 (−2.12,−1.71)	< 0.001	−0.65 (−0.74,−0.55)	< 0.001
Daily	−2.30 (−2.50,−2.10)	< 0.001	−0.90 (−0.99,−0.81)	< 0.001
**Frequency of yogurt (Ref**. = **Never)**				
Less than once per month	−0.74 (−0.90,−0.58)	< 0.001	−0.09 (−0.16,−0.02)	0.016
1–3 days/week	−1.12 (−1.27,−0.96)	< 0.001	−0.40 (−0.47,−0.34)	< 0.001
4–6 days/week	−0.80 (−0.99,−0.62)	< 0.001	−0.23 (−0.32,−0.15)	< 0.001
Daily	−0.88 (−1.09,−0.68)	< 0.001	−0.35 (−0.45,−0.26)	< 0.001
**Frequency of milk powder (Ref**. = **Never)**				
Less than once per month	0.12 (−0.03, 0.28)	0.117	0.02 (−0.05, 0.09)	0.610
1–3 days/week	0.25 (0.09, 0.42)	0.003	−0.28 (−0.36,−0.21)	< 0.001
4–6 days/week	0.41 (0.16, 0.66)	0.001	−0.19 (−0.31,−0.08)	< 0.001
Daily	0.69 (0.42, 0.96)	< 0.001	−0.21 (−0.33,−0.08)	0.001
**Frequency of cheese/cheese cubes (Ref**. = **Never)**				
Less than once per month	0.12 (−0.06, 0.29)	0.195	0.12 (0.04, 0.20)	0.003
1–3 days/week	0.28 (0.07, 0.49)	0.009	−0.17 (−0.27,−0.07)	< 0.001
4–6 days/week	0.57 (0.26, 0.87)	< 0.001	−0.21 (−0.35,−0.07)	0.003
Daily	0.95 (0.60, 1.30)	< 0.001	−0.68 (−0.84,−0.52)	< 0.001
**Daily amount of milk, yogurt, and milk powder (200 ml)**	0.10 (0.03, 0.17)	0.004	0.20 (0.17, 0.23)	< 0.001
**Daily amount of cheese/cheese cubes (Ref**. = **Never)**				
1–3 pieces	−0.31 (−0.49,−0.14)	< 0.001	−0.05 (−0.13, 0.03)	0.208
4–6 pieces	0.12 (−0.10, 0.35)	0.287	−0.12 (−0.23,−0.02)	0.020
7–9 pieces	0.91 (0.59, 1.24)	< 0.001	0.00 (−0.15, 0.15)	0.991
10–12 pieces	1.06 (0.68, 1.45)	< 0.001	−0.01 (−0.19, 0.16)	0.897
13–15 pieces	2.09 (1.50, 2.68)	< 0.001	0.33 (0.06, 0.60)	0.016
>15 pieces	1.67 (1.06, 2.28)	< 0.001	0.73 (0.45, 1.00)	< 0.001

BMI, body mass index; CI, confidence interval.

Estimates for milk powder and cheese/cheese cube intake were more heterogeneous. For depressive symptoms, daily milk powder intake (β = 0.69, 95% CI: 0.42 to 0.96) and daily cheese/cheese cube intake (β = 0.95, 95% CI: 0.60 to 1.30) showed positive estimates in the mutually adjusted model; their corresponding estimates in the single-exposure models were 0.60 (95% CI: 0.40 to 0.80) and 0.77 (95% CI: 0.54 to 1.01). For anxiety symptoms, however, the inverse estimates for these exposures were substantially attenuated after mutual adjustment. For example, the estimate for daily milk powder intake changed from−0.92 (95% CI:−1.01 to −0.82) in the single-exposure model to −0.21 (95% CI:−0.33 to −0.08) in the mutually adjusted model, while the estimate for daily cheese/cheese cube intake changed from−1.21 (95% CI:−1.32 to −1.11) to −0.68 (95% CI:−0.84 to -0.52).

The clearest change in direction across model specifications was observed for the combined daily amount of milk, yogurt, and milk powder in relation to depressive symptoms. Each additional 200-ml unit corresponded to a small inverse estimate in the single-exposure model (β = −0.12, 95% CI:−0.18 to −0.05), but a small positive estimate after mutual adjustment for the other dairy variables (β = 0.10, 95% CI: 0.03 to 0.17). Given the small magnitude of both estimates, this reversal was considered model-dependent rather than evidence of a substantively large association. For anxiety symptoms, the estimate remained positive but increased from β = 0.08 (95% CI: 0.06 to 0.11) in the single-exposure model to β = 0.20 (95% CI: 0.17 to 0.23) in the mutually adjusted model. For cheese/cheese cube amount, higher intake categories showed more appreciable positive estimates for depressive symptoms, particularly 13–15 pieces/day (β = 2.09, 95% CI: 1.50 to 2.68) and >15 pieces/day (β = 1.67, 95% CI: 1.06 to 2.28). For anxiety symptoms, estimates at lower cheese amounts were close to the null after mutual adjustment, whereas the highest category (>15 pieces/day) corresponded to a 0.73-point higher score (95% CI: 0.45 to 1.00).

Model diagnostics indicated modest explanatory performance of the primary linear regression models (adjusted R^2^ = 0.111 for depressive symptoms and 0.103 for anxiety symptoms). Only 1.84% and 2.05% of observations had absolute standardized residuals greater than 3, respectively, and maximum Cook's distances were small (< 0.002), suggesting no strongly influential individual observations. Detailed model diagnostics are presented in Supplementary Table S3.

Results from models in which each dairy variable was entered separately are shown in Supplementary Table S4 for depressive symptoms and Supplementary Table S5 for anxiety symptoms. In sensitivity analyses using MI, the associations between dairy intake variables and depressive and anxiety symptoms were largely consistent with those observed in the main complete-case analyses. The direction and magnitude of the regression coefficients remained similar after imputing missing dairy intake variables and covariates. Detailed results from the MI analyses are presented in Supplementary Table S6. The findings were also broadly robust to alternative outcome specifications. Negative binomial regression models of CES-D and GAD-7 scores yielded generally similar patterns to the primary linear regression analyses, particularly for the inverse associations of milk and yogurt intake frequencies with depressive and anxiety symptoms. Logistic regression analyses using dichotomized elevated depressive and anxiety symptoms also showed broadly consistent associations, although more heterogeneous patterns remained for milk powder, cheese/cheese cubes, and total dairy intake amount (Supplementary Table S7).

### 3.3 Trend analyses

Adjusted trend analyses are presented in Table 4. The largest inverse trend estimates were observed for milk intake frequency, with β = −1.95 (95% CI:−2.11 to −1.79) for depressive symptoms and β = −0.91 (95% CI:−0.98 to −0.84) for anxiety symptoms. Yogurt intake frequency also showed inverse trends (depressive symptoms: β = −0.87, 95% CI:−1.03 to −0.71; anxiety symptoms: β = −0.86, 95% CI:−0.93 to −0.78). In contrast, higher milk powder and cheese/cheese cube frequencies showed positive trend estimates for depressive symptoms (β = 0.58, 95% CI: 0.40 to 0.77 and β = 0.43, 95% CI: 0.25 to 0.61, respectively) but inverse estimates for anxiety symptoms (β = −0.92, 95% CI:−1.00 to −0.83 and β = −0.82, 95% CI:−0.90 to −0.73, respectively).

**Table 4:** Adjusted trend analyses of dairy intake variables with depressive and anxiety symptoms.

**Outcome**	Dairy intake	Beta (95% CI)	*P* for trend	FDR-adjusted *P* for trend	Overall *P*
Depressive symptoms	Frequency of milk	−1.95 (−2.11, −1.79)	< 0.001	< 0.001	< 0.001
Depressive symptoms	Frequency of yogurt	−0.87 (−1.03, −0.71)	< 0.001	< 0.001	< 0.001
Depressive symptoms	Frequency of milk powder	0.58 (0.40, 0.77)	< 0.001	< 0.001	< 0.001
Depressive symptoms	Frequency of cheese/cheese cubes	0.43 (0.25, 0.61)	< 0.001	< 0.001	< 0.001
Depressive symptoms	Daily amount of milk, yogurt, and milk powder (200 ml)	−0.12 (−0.18, −0.05)	< 0.001	< 0.001	< 0.001
Depressive symptoms	Daily amount of cheese/cheese cubes	0.46 (0.27, 0.64)	< 0.001	< 0.001	< 0.001
Anxiety symptoms	Frequency of milk	−0.91 (−0.98, −0.84)	< 0.001	< 0.001	< 0.001
Anxiety symptoms	Frequency of yogurt	−0.86 (−0.93, −0.78)	< 0.001	< 0.001	< 0.001
Anxiety symptoms	Frequency of milk powder	−0.92 (−1.00, −0.83)	< 0.001	< 0.001	< 0.001
Anxiety symptoms	Frequency of cheese/cheese cubes	−0.82 (−0.90, −0.73)	< 0.001	< 0.001	< 0.001
Anxiety symptoms	Daily amount of milk, yogurt, and milk powder (200 ml)	0.08 (0.06, 0.11)	< 0.001	< 0.001	< 0.001
Anxiety symptoms	Daily amount of cheese/cheese cubes	−0.59 (−0.68, −0.51)	< 0.001	< 0.001	< 0.001

CI, confidence interval; FDR, false discovery rate. Each dairy exposure was modeled separately and adjusted for age, gender, ethnicity, education level, region, body mass index, living on campus, history of smoking, history of drinking, physical inactivity, low fruit or vegetable intake, unhealthy diet, father's education level, and mother's education level.

Trend estimates for the combined daily amount of milk, yogurt, and milk powder were small in magnitude (depressive symptoms: β = −0.12, 95% CI:−0.18 to −0.05 per 200-ml unit; anxiety symptoms: β = 0.08, 95% CI: 0.06 to 0.11 per 200-ml unit), despite their statistical precision in this large sample. Higher ordered categories of cheese/cheese cube amount showed a positive trend for depressive symptoms (β = 0.46, 95% CI: 0.27 to 0.64) and an inverse trend for anxiety symptoms (β = −0.59, 95% CI:−0.68 to −0.51). All 12 trend tests remained below the prespecified false discovery rate threshold after Benjamini-Hochberg correction (all FDR-adjusted *P* for trend < 0.001). Interpretation therefore focused primarily on the magnitude, direction, and confidence intervals of the estimates rather than statistical significance alone.

### 3.4 RCS analyses

RCS analyses were further performed to examine potential nonlinear associations between dairy intake variables and depressive or anxiety symptom scores. The RCS curves from the simultaneous dairy models are shown in Supplementary Figure S2 for depressive symptoms and Supplementary Figure S3 for anxiety symptoms. Overall, the spline patterns were broadly consistent with the multivariable regression results. The daily amount of milk, yogurt, and milk powder tended to show a weak positive association with depressive symptom scores after simultaneous adjustment for other dairy variables. The RCS analysis indicated that the association between total daily amount of milk, yogurt, and milk powder intake and depressive symptoms was nonlinear. The predicted depressive symptom score reached the lowest point at approximately 1.5 units of 200 ml/day (around 300 ml/day) in both the simultaneous dairy model (Supplementary Figure S2) and the separate dairy model (Supplementary Figure S4). The supplementary RCS curves from separate dairy models are shown in Supplementary Figures S4, S5.

### 3.5 Interaction analyses

Exploratory interaction analyses are presented in Supplementary Table S8. For depressive symptoms, evidence of interaction with gender remained for all six dairy intake variables after FDR correction (all FDR-adjusted *P* for interaction ≤ 0.002). Evidence of regional heterogeneity was observed for milk frequency (FDR-adjusted *P* = 0.001), milk powder frequency (*P* = 0.020), cheese/cheese cube frequency (*P* = 0.016), the combined daily amount of milk, yogurt, and milk powder (*P* = 0.004), and cheese/cheese cube amount (*P* = 0.022), whereas little evidence was observed for yogurt frequency (*P* = 0.629).

For anxiety symptoms, FDR-adjusted interaction tests provided evidence of gender-related heterogeneity for milk, yogurt, milk powder, and cheese/cheese cube frequencies and for cheese/cheese cube amount (all FDR-adjusted *P* for interaction < 0.001), but not for the combined daily amount of milk, yogurt, and milk powder (*P* = 0.171). Regional heterogeneity was observed for milk frequency (*P* < 0.001), cheese/cheese cube frequency (*P* < 0.001), the combined dairy amount (*P* = 0.049), and cheese/cheese cube amount (*P* < 0.001), but not for yogurt frequency (*P* = 0.389) or milk powder frequency (*P* = 0.264). These exploratory findings indicate that heterogeneity by gender and region was not uniform across dairy measures or mental health outcomes and should therefore be interpreted cautiously. To further illustrate the magnitude and direction of these subgroup differences, subgroup-specific estimates with 95% CIs are presented for girls and boys in Supplementary Table S9 and for suburban and urban students in Supplementary Table S10.

### 3.6 School-level mixed-effects sensitivity analyses

School-level random-intercept models generally showed similar patterns to the primary linear regression analyses, particularly for the inverse associations of milk and yogurt intake frequencies with depressive and anxiety symptoms (Supplementary Table S11). Some weaker associations were attenuated after accounting for school-level clustering; notably, the association between the combined daily amount of milk, yogurt, and milk powder and depressive symptoms was close to the null (β = −0.01, 95% CI:−0.08 to 0.05). The school-level mixed-effects interaction analyses also showed that most gender-related interactions were broadly consistent with the primary exploratory interaction analyses, whereas evidence for several regional interactions was attenuated after accounting for school-level clustering (Supplementary Table S12).

## 4 Discussion

This study systematically examined the associations of six dairy intake variables with depressive and anxiety symptoms among 135,138 middle school students. The main findings were as follows. First, higher frequencies of milk and yogurt intake were generally associated with lower depressive and anxiety symptom scores. Second, the results for milk powder- and cheese-related variables were more complex, showing inconsistent associations across different outcomes and intake levels. Third, estimates for the combined daily amount of milk, yogurt, and milk powder were sensitive to model specification, particularly for depressive symptoms. Fourth, exploratory analyses suggested that the associations between several dairy intake variables and depressive or anxiety symptoms may vary by gender and urban-suburban context. Overall, these findings suggest that the relationship between dairy intake and mental health among adolescents may vary by dairy type, intake level, and population subgroup. Depressive and anxiety symptom scores were strongly correlated in this sample. Therefore, broadly similar association patterns across the two outcomes should be viewed as partly overlapping evidence rather than as fully independent replication.

In the present study, depressive and anxiety symptom scores were higher among girls, students in higher grades, those with a history of smoking or alcohol use, and those with physical inactivity, low fruit and vegetable intake, or unhealthy dietary behaviors. This finding is broadly consistent with previous studies on risk factors for adolescent mental health problems (5, 26, 27). Higher levels of depressive and anxiety symptoms among adolescent girls may be related to pubertal development, sex hormone changes, emotion regulation patterns, body image pressure, and gendered social expectations (28, 29). Meanwhile, behavioral factors such as smoking, alcohol use, unhealthy dietary behaviors, and physical inactivity may not only be consequences of psychological distress but may also influence mental health through pathways related to inflammation, sleep, energy metabolism, and stress-coping patterns (30).

This study also showed a clear imbalance in dairy intake in this sample. Although approximately one-quarter of students consumed milk daily, a proportion of students rarely consumed milk. The proportion of daily yogurt intake was relatively low, while milk powder and cheese/cheese cubes were consumed even less frequently. In comparison with previous evidence, the proportion of students who consumed milk daily in the present study was 27.5%, which was higher than the 22.5% of Chinese children and adolescents aged 6–17 years who consumed fluid milk at least once per day in the 2010–2012 China National Nutrition and Health Surveillance, but still far from universal daily intake (31). In that national survey, 41.6% of children and adolescents reported no fluid milk intake, whereas only 7.3% of students in the present study reported never drinking milk, suggesting that milk accessibility and acceptance may have improved in recent years (31). For yogurt, however, the daily intake proportion in the present study was 10.5%, slightly lower than the 13.6% reported for daily yogurt consumption in the national survey, indicating that yogurt intake remained less regular than milk intake in this sample (31). Regarding intake amount, the mean amount of milk, yogurt, and milk powder in the present study was approximately 234 ml/day, which was higher than the average fluid milk and yogurt intakes reported in earlier Chinese children and adolescents (31). Evidence from a recent study conducted in two Chinese provinces also showed that milk remained the dominant source of dairy intake among Chinese children, with UHT milk accounting for 78.7% of total dairy intake, whereas yogurt, milk powder, and cheese accounted for only 9.8%, 1.3%, and 2.6%, respectively (32). This pattern is broadly consistent with the present finding that milk powder and cheese/cheese cubes were much less commonly consumed than milk and yogurt. The remaining differences between the present findings and previous estimates may be related to differences in survey year, study region, age composition, urban-suburban distribution, and dietary assessment methods. From a public health perspective, insufficient dairy intake may indicate inadequate intake of high-quality protein, calcium, and other micronutrients among some adolescents. However, the present study also suggests that the associations between high frequency or high amount of certain dairy products and mental health symptoms are not entirely linear.

In multivariable regression analyses, the frequencies of milk and yogurt intake showed relatively stable associations with lower depressive and anxiety symptom scores. Previous research among Chinese children and adolescents has also found that habitual dairy consumption is associated with lower depressive and social anxiety symptoms (17). Several biological pathways have been proposed in the literature, including nutrient-related neurotransmitter metabolism and, for fermented dairy products, microbiota–gut–brain signaling (15, 33–40). However, none of these mechanisms was directly measured in the present study, and they should therefore be regarded only as possible explanations rather than mechanisms demonstrated by our data.

The comparison between the single-exposure and mutually adjusted models provides additional context for interpreting these findings. Associations for milk frequency were highly stable across the two specifications, whereas those for yogurt, milk powder, cheese/cheese cubes, and particularly the combined daily amount of milk, yogurt, and milk powder were more sensitive to mutual adjustment. Importantly, both estimates were small in absolute magnitude, suggesting that the apparent reversal should not be interpreted as evidence for a clinically meaningful adverse effect of higher dairy intake. Such changes are plausible because the two model specifications estimate different conditional associations. This distinction is particularly relevant for the combined daily amount of milk, yogurt, and milk powder, because this measure is intrinsically related to the frequency variables for the same products. Mutual adjustment for correlated and partly overlapping dietary indicators can therefore redistribute shared variation among coefficients and produce attenuation or suppression, including changes in direction. Similar attenuation was observed for the inverse associations of milk powder and cheese/cheese cube frequency with anxiety symptoms after mutual adjustment. Findings that were stable across specifications, particularly those for milk frequency, were therefore interpreted with greater confidence, whereas model-dependent estimates, especially those for total dairy amount, were interpreted cautiously. These conditional associations should not be interpreted as causal substitution effects between dairy products.

In addition, the complex findings for milk powder and cheese/cheese cubes deserve attention. Compared with plain milk and yogurt, milk powder and cheese/cheese cubes may have more heterogeneous consumption contexts in adolescents' daily diets. In particular, cheese/cheese cubes among Chinese adolescents may often have snack-like, sweetened, and processed-food characteristics, and their intake may also reflect higher energy intake, preference for sweet snacks, or other unhealthy dietary behaviors. Therefore, the associations between cheese-related variables and higher mental health symptom scores may not necessarily indicate an adverse effect of traditional dairy products themselves, but may instead be related to processing form, nutrient composition, and the overall dietary pattern.

Restricted cubic spline analyses further supported a cautious interpretation of the dose-response relationships. For the association between total daily amount of milk, yogurt, and milk powder intake and depressive symptoms, the RCS analyses suggested a nonlinear pattern, with the lowest predicted symptom score observed at approximately 1.5 units of 200 ml/day (around 300 ml/day) in both the simultaneous dairy model and the separate dairy model. This observed point was close to the recommended daily dairy intake for school-aged children in the Chinese dietary guidelines (41). However, this point represents a descriptive feature of the fitted curve rather than an estimated optimal intake threshold. Because uncertainty around the location of the minimum was not quantified and the study was cross-sectional, no specific intake level should be inferred as optimal from this analysis.

Exploratory interaction analyses suggested that the associations between dairy intake and mental health symptoms may vary by gender and urban-suburban context. For several dairy frequency measures, the inverse associations with depressive or anxiety symptoms appeared weaker among boys than among girls, whereas some positive associations were more pronounced among boys. An exception was the combined daily amount of milk, yogurt, and milk powder in relation to depressive symptoms, for which the interaction estimate with boys was negative. Regional heterogeneity was also observed for several dairy measures, although the patterns were not consistent across exposures or outcomes. These subgroup differences may reflect variation in dietary preferences, weight-control concerns, body image, pubertal development, family food environments, dairy product availability, and school-related lifestyle patterns across gender and residential contexts (42–47). Nevertheless, because these interaction analyses were exploratory and involved multiple comparisons, the findings should be regarded as hypothesis-generating and interpreted cautiously despite FDR correction.

This study has several strengths. First, the large sample size, covering students of different genders, educational stages, and urban-suburban backgrounds, allowed relatively stable estimation of the associations between dairy intake and mental health symptoms. Second, rather than examining only overall dairy intake, this study distinguished among milk, yogurt, milk powder, cheese/cheese cubes, and intake amount, which helped reveal differences across dairy types. Third, this study used multivariable regression, RCS models, and interaction analyses, providing relatively comprehensive evidence from the perspectives of linear associations, nonlinear trends, and population heterogeneity.

Several limitations should also be noted. First, the cross-sectional design precludes causal inference, and students with higher depressive or anxiety symptoms may have changed their dietary habits; therefore, reverse causality cannot be ruled out. Second, dairy intake and mental health symptoms were both assessed using self-reported questionnaires, which may be subject to recall bias and reporting bias. Third, although the models adjusted for multiple demographic, behavioral, and family-related factors, residual confounding from unmeasured factors cannot be excluded. Several potentially important factors associated with adolescent mental health, including parent-child relationships, bullying victimization, sleep duration, screen time, academic or after-school stress, socioeconomic circumstances, family structure, certain health conditions, lactose intolerance, and cow's milk protein allergy, were not included in the present analyses. Future studies should consider incorporating these factors to further reduce potential confounding bias and better clarify the associations between dairy intake and adolescent mental health. Fourth, the assessment and reconstruction of dairy exposure were constrained by the structure of the original surveillance questionnaire. The available data did not permit all dairy products to be converted into a common quantitative metric such as grams or milliliters per day. Specifically, the continuous amount variable combined milk, yogurt, and milk powder rather than recording the daily amount of each product separately, while cheese/cheese cubes were recorded only as numbers of pieces without corresponding gram weights. In addition, the available data did not provide sufficient information to derive product-specific quantities for milk powder or to distinguish conventional cheese from cheese cubes. Any *post hoc* conversion into a common metric would therefore require assumptions about product preparation and portion size that were not supported by the original data and could introduce additional measurement error. Consequently, a harmonized estimate of total dairy intake, separate continuous dose–response analyses for individual dairy products, and formal substitution analyses between dairy products could not be reliably performed. The six dairy variables should therefore be interpreted as questionnaire-derived indicators of different dairy consumption patterns rather than as directly interchangeable quantitative measures, and the simultaneous models should not be interpreted as estimating product-substitution effects. Total energy intake was also unavailable because the surveillance questionnaire was not designed to quantify overall dietary intake. More broadly, dairy intake was assessed using a standardized surveillance questionnaire rather than a comprehensive dietary assessment instrument such as repeated 24-h dietary recalls or a validated food-frequency questionnaire, and measurement error and exposure misclassification cannot be excluded. Future studies using detailed product-specific intake data, standardized portion weights, and comprehensive dietary assessment are needed to quantify dairy intake on a common scale and to evaluate total, product-specific, and substitution associations more rigorously. Fifth, given the large sample size, even small differences could reach conventional levels of statistical significance. Therefore, the magnitude and precision of the estimated associations, rather than *P* values alone, should guide interpretation. Finally, although the final analytic sample was large, exclusion due to missing information on all dairy intake items and parental education variables may have introduced selection bias. Participants excluded from the analysis differed from those included in several demographic characteristics. In addition, although the original surveillance followed a multistage stratified cluster sampling design, sampling weights were not provided in the secondary analytic dataset, and the complete sampling probabilities required to reconstruct valid individual-level weights across all sampling stages were unavailable. Classroom identifiers and corresponding classroom-level sampling information were also not retained, precluding a complete reconstruction of the original hierarchical clustering structure. These limitations may affect the representativeness and generalizability of the findings, which should therefore be interpreted cautiously when extrapolating to other student populations or regions.

Despite these limitations, the present study may provide useful context for future research on adolescent diet and mental health. The relatively consistent inverse associations observed for milk and yogurt frequency, together with the more heterogeneous findings for milk powder, cheese/cheese cubes, and amount-based measures, suggest that dairy intake may be better characterized using multiple dimensions rather than a single overall measure. These findings also highlight the importance of distinguishing dairy type, intake frequency, and intake amount in future studies. However, because the present study was cross-sectional and residual confounding and reverse causality cannot be excluded, the observed associations should not be used to infer that changing dairy intake would improve or worsen adolescent mental health. Longitudinal studies and studies with more detailed product-specific dietary assessment are needed to clarify the temporal and potentially causal nature of these associations.

## 5 Conclusion

In this large cross-sectional sample of middle school students, dairy intake variables showed heterogeneous associations with depressive and anxiety symptoms. More frequent milk and yogurt intake was relatively consistently associated with lower symptom scores, whereas associations for milk powder, cheese/cheese cubes, and amount-based dairy measures were more heterogeneous and, for some measures, sensitive to model specification. Exploratory analyses also suggested potential heterogeneity by gender and urban-suburban context, although these patterns were not consistent across all dairy measures or outcomes. Given the cross-sectional observational design, these findings do not establish causal effects or optimal dairy intake patterns. Further longitudinal studies with detailed product-specific dietary assessment are needed to clarify the temporal relationships between dairy consumption and adolescent mental health.

## Data Availability

The data used in this study were obtained from the provincial surveillance system on student health and contain sensitive information on adolescents. In accordance with ethical requirements and data protection regulations, the datasets are not publicly available. De-identified data may be accessed upon reasonable request for research purposes. Interested researchers may contact the author, Dr. Lianlong Yu (Email: lianlong00a@163.com), at the Shandong Center for Disease Control and Prevention. Access to the data requires on-site review and analysis at the Shandong CDC in compliance with institutional and provincial ethical guidelines.
