# Supplementary material for: Type-, dose-, and subgroup-specific associations of dairy intake with depressive and anxiety symptoms among middle school students
Source: Front Nutr. 2026 Oct 13;13:1954062. doi: 10.3389/fnut.2026.1954062 (PMC13710028; doi:10.3389/fnut.2026.1954062)
Supplement: Supplementary file 1 [file Table_1.docx]

**Table S1. Comparison of characteristics between included and excluded participants**

|  | Included | Excluded | Statistic | P value |
| --- | --- | --- | --- | --- |
| Number | 135,138 | 21,143 |  |  |
| Age (mean ± SD) | 14.41 ± 1.87 | 14.19 ± 1.91 | F = 260.72 | < 0.001 |
| Gender |  |  | χ² = 2.64 | 0.104 |
| Boys | 70,708 (52.3) | 11,190 (52.9) |  |  |
| Girls | 64,430 (47.7) | 9,953 (47.1) |  |  |
| Ethnicity |  |  | χ² = 0.07 | 0.796 |
| Han | 133,141 (98.5) | 20,836 (98.5) |  |  |
| Other | 1,997 (1.5) | 307 (1.5) |  |  |
| Education level |  |  | χ² = 141.8 | < 0.001 |
| Junior high school | 74,827 (55.4) | 12,632 (59.7) |  |  |
| Senior high school | 60,311 (44.6) | 8,511 (40.3) |  |  |
| Region |  |  | χ² = 619.7 | < 0.001 |
| Urban | 68,036 (50.3) | 8,698 (41.1) |  |  |
| Suburban | 67,102 (49.7) | 12,445 (58.9) |  |  |
| BMI (mean ± SD) | 22.17 ± 4.75 | 22.07 ± 4.72 | F=9.46 | 0.002 |
| Living on campus |  |  | χ²=9.31 | 0.002 |
| Yes | 48,791 (36.1) | 7,404 (35.0) |  |  |
| No | 86,347 (63.9) | 13,739 (65.0) |  |  |
| History of smoking |  |  | χ²=25.89 | < 0.001 |
| Yes | 3,919 (2.9) | 481 (2.3) |  |  |
| No | 131,206 (97.1) | 20,662 (97.7) |  |  |
| Missing | 13 | 0 |  |  |
| History of drinking |  |  | χ²=339.19 | < 0.001 |
| Yes | 17,182 (12.7) | 1,748 (8.3) |  |  |
| No | 117,956 (87.3) | 19,395 (91.7) |  |  |
| Physical inactivity |  |  | χ²=27.46 | < 0.001 |
| Yes | 118,292 (87.5) | 18,777 (88.8) |  |  |
| No | 16,846 (12.5) | 2,366 (11.2) |  |  |
| Low fruit or vegetable intake |  |  | χ²=14.58 | < 0.001 |
| Yes | 51,689 (38.2) | 8,378 (39.6) |  |  |
| No | 83,449 (61.8) | 12,765 (60.4) |  |  |
| Unhealthy diet |  |  | χ²=13.06 | < 0.001 |
| Yes | 16,877 (12.5) | 2,454 (11.6) |  |  |
| No | 118,255 (87.5) | 18,689 (88.4) |  |  |
| Missing | 6 | 0 |  |  |
| Depressive symptoms | 9.83 ± 8.72 | 9.41 ± 8.29 | F=43.29 | < 0.001 |
| Anxiety symptoms | 2.87 ± 3.96 | 2.54 ± 3.64 | F=124.27 | < 0.001 |

Note: BMI, body mass index; SD, standard deviation.

**Table S2. Univariate associations of dairy intake variables with depressive and anxiety symptoms**

| Variables | Depressive symptoms | | | Anxiety symptoms | | |
| --- | --- | --- | --- | --- | --- | --- |
|  | Mean ± SD | F/r | P | Mean ± SD | F/r | P |
| Frequency of milk |  | 553.909 | <0.001 |  | 545.442 | <0.001 |
| Never | 12.57 ± 10.46 |  |  | 3.97 ± 4.83 |  |  |
| Less than once per month | 11.69 ± 9.69 |  |  | 3.76 ± 4.30 |  |  |
| 1-3 days/week | 9.67 ± 8.22 |  |  | 2.78 ± 3.76 |  |  |
| 4-6 days/week | 9.42 ± 8.54 |  |  | 2.82 ± 3.85 |  |  |
| Daily | 8.87 ± 8.25 |  |  | 2.36 ± 3.76 |  |  |
| Frequency of yogurt |  | 200.869 | <0.001 |  | 266.919 | <0.001 |
| Never | 11.24 ± 9.83 |  |  | 3.34 ± 4.45 |  |  |
| Less than once per month | 10.13 ± 9.09 |  |  | 3.25 ± 4.01 |  |  |
| 1-3 days/week | 9.21 ± 8.08 |  |  | 2.61 ± 3.68 |  |  |
| 4-6 days/week | 9.89 ± 8.74 |  |  | 2.91 ± 3.99 |  |  |
| Daily | 9.62 ± 8.32 |  |  | 2.32 ± 3.98 |  |  |
| Frequency of milk powder |  | 12.384 | <0.001 |  | 248.149 | <0.001 |
| Never | 9.85 ± 8.94 |  |  | 3.03 ± 4.00 |  |  |
| Less than once per month | 9.72 ± 8.73 |  |  | 3.05 ± 3.93 |  |  |
| 1-3 days/week | 9.51 ± 7.55 |  |  | 2.27 ± 3.58 |  |  |
| 4-6 days/week | 10.28 ± 8.55 |  |  | 2.54 ± 4.10 |  |  |
| Daily | 10.16 ± 8.13 |  |  | 1.91 ± 3.92 |  |  |
| Frequency of cheese/cheese cubes |  | 39.943 | <0.001 |  | 269.831 | <0.001 |
| Never | 9.92 ± 8.95 |  |  | 3.02 ± 4.00 |  |  |
| Less than once per month | 9.49 ± 8.66 |  |  | 3.04 ± 3.89 |  |  |
| 1-3 days/week | 9.39 ± 7.56 |  |  | 2.28 ± 3.61 |  |  |
| 4-6 days/week | 10.69 ± 8.68 |  |  | 2.57 ± 4.19 |  |  |
| Daily | 10.43 ± 8.10 |  |  | 1.62 ± 3.93 |  |  |
| Daily amount of milk, yogurt, and milk powder (200 mL units) |  | -0.034 | <0.001 |  | -0.006 | 0.040 |
| Daily amount of cheese/cheese cubes |  | 61.972 | <0.001 |  | 74.394 | <0.001 |
| Never | 9.94 ± 8.95 |  |  | 3.02 ± 4.00 |  |  |
| 1-3 pieces | 9.20 ± 8.11 |  |  | 2.58 ± 3.75 |  |  |
| 4-6 pieces | 9.73 ± 7.84 |  |  | 2.41 ± 3.67 |  |  |
| 7-9 pieces | 10.82 ± 8.40 |  |  | 2.66 ± 4.13 |  |  |
| 10-12 pieces | 11.00 ± 9.10 |  |  | 2.57 ± 4.35 |  |  |
| 13-15 pieces | 12.25 ± 9.68 |  |  | 3.25 ± 5.15 |  |  |
| >15 pieces | 12.06 ± 11.04 |  |  | 3.34 ± 5.28 |  |  |

Note: SD, standard deviation.

**Table S3. Diagnostic statistics for the primary multivariable linear regression models**

| Diagnostic | Depressive symptoms | Anxiety symptoms |
| --- | --- | --- |
| R² | 0.112 | 0.103 |
| Adjusted R² | 0.111 | 0.103 |
| RMSE | 8.21 | 3.75 |
| MAE | 6.02 | 2.8 |
| Absolute standardized residuals >3, % | 1.84 | 2.05 |
| Maximum Cook’s distance | 0.001 | 0.001 |
| Breusch-Pagan test, χ² | 3697.09 | 3427.12 |
| P value for Breusch-Pagan test | <0.001 | <0.001 |
| Ramsey RESET statistic | 9.62 | 59.58 |
| P value for Ramsey RESET test | <0.001 | <0.001 |

Note: R², coefficient of determination; RMSE, root mean square error; MAE, mean absolute error. The Breusch–Pagan test was used to assess heteroscedasticity, and the Ramsey RESET test was used to assess potential functional-form misspecification. Cook’s distance and standardized residuals were used to evaluate influential observations and extreme residuals, respectively.

**Table S4. Associations between individual dairy intake variables and depressive symptoms in the single-exposure covariate-adjusted models**

| **Variable** | **Model D1 beta (95% CI)** | **Model D1 P** | **Model D2 beta (95% CI)** | **Model D2 P** | **Model D3 beta (95% CI)** | **Model D3 P** | **Model D4 beta (95% CI)** | **Model D4 P** | **Model D5 beta (95% CI)** | **Model D5 P** | **Model D6 beta (95% CI)** | **Model D6 P** |
| --- | --- | --- | --- | --- | --- | --- | --- | --- | --- | --- | --- | --- |
| Frequency of milk (Ref. = Never) | | | |  |  |  |  |  |  |  |  |  |
| Less than once per month | -0.65 (-0.86, -0.44) | <0.001 |  |  |  |  |  |  |  |  |  |  |
| 1-3 days/week | -2.05 (-2.23, -1.87) | <0.001 |  |  |  |  |  |  |  |  |  |  |
| 4-6 days/week | -1.97 (-2.16, -1.78) | <0.001 |  |  |  |  |  |  |  |  |  |  |
| Daily | -2.31 (-2.50, -2.13) | <0.001 |  |  |  |  |  |  |  |  |  |  |
| Frequency of yogurt (Ref. = Never) | | | |  |  |  |  |  |  |  |  |  |
| Less than once per month |  |  | -0.85 (-1.00, -0.70) | <0.001 |  |  |  |  |  |  |  |  |
| 1-3 days/week |  |  | -1.40 (-1.54, -1.26) | <0.001 |  |  |  |  |  |  |  |  |
| 4-6 days/week |  |  | -0.78 (-0.95, -0.61) | <0.001 |  |  |  |  |  |  |  |  |
| Daily |  |  | -0.97 (-1.15, -0.78) | <0.001 |  |  |  |  |  |  |  |  |
| Frequency of milk powder (Ref. = Never) | | | |  |  |  |  |  |  |  |  |  |
| Less than once per month |  |  |  |  | 0.06 (-0.09, 0.21) | 0.441 |  |  |  |  |  |  |
| 1-3 days/week |  |  |  |  | 0.03 (-0.12, 0.17) | 0.716 |  |  |  |  |  |  |
| 4-6 days/week |  |  |  |  | 0.81 (0.60, 1.03) | <0.001 |  |  |  |  |  |  |
| Daily |  |  |  |  | 0.60 (0.40, 0.80) | <0.001 |  |  |  |  |  |  |
| Frequency of cheese/cheese cubes (Ref. = Never) | | | |  |  |  |  |  |  |  |  |  |
| Less than once per month |  |  |  |  |  |  | -0.11 (-0.23, 0.01) | 0.067 |  |  |  |  |
| 1-3 days/week |  |  |  |  |  |  | -0.01 (-0.15, 0.13) | 0.843 |  |  |  |  |
| 4-6 days/week |  |  |  |  |  |  | 1.03 (0.80, 1.27) | <0.001 |  |  |  |  |
| Daily |  |  |  |  |  |  | 0.77 (0.54, 1.01) | <0.001 |  |  |  |  |
| Daily amount of milk, yogurt, and milk powder (200 mL units) |  |  |  |  |  |  |  |  | -0.12 (-0.18, -0.05) | <0.001 |  |  |
| Daily amount of cheese/cheese cubes (Ref. = Never) | | | | |  |  |  |  |  |  |  |  |
| 1-3 pieces |  |  |  |  |  |  |  |  |  |  | -0.29 (-0.40, -0.18) | <0.001 |
| 4-6 pieces |  |  |  |  |  |  |  |  |  |  | 0.17 (-0.01, 0.34) | 0.058 |
| 7-9 pieces |  |  |  |  |  |  |  |  |  |  | 1.13 (0.84, 1.41) | <0.001 |
| 10-12 pieces |  |  |  |  |  |  |  |  |  |  | 1.42 (1.09, 1.76) | <0.001 |
| 13-15 pieces |  |  |  |  |  |  |  |  |  |  | 2.63 (2.06, 3.20) | <0.001 |
| >15 pieces |  |  |  |  |  |  |  |  |  |  | 2.21 (1.62, 2.80) | <0.001 |

Note: Each dairy intake variable was entered separately. Models were adjusted for age, gender, ethnicity, education level, region, body mass index, living on campus, history of smoking, history of drinking, physical inactivity, low fruit or vegetable intake, unhealthy diet, father’s education level, and mother’s education level.

**Table S5. Associations between individual dairy intake variables and anxiety symptoms in the single-exposure covariate-adjusted models**

| **Variable** | **Model A1 beta (95% CI)** | **Model A1 P** | **Model A2 beta (95% CI)** | **Model A2 P** | **Model A3 beta (95% CI)** | **Model A3 P** | **Model A4 beta (95% CI)** | **Model A4 P** | **Model A5 beta (95% CI)** | **Model A5 P** | **Model A6 beta (95% CI)** | **Model A6 P** |
| --- | --- | --- | --- | --- | --- | --- | --- | --- | --- | --- | --- | --- |
| Frequency of milk (Ref. = Never) |  |  |  |  |  |  |  |  |  |  |  |  |
| Less than once per month | -0.12 (-0.22, -0.03) | 0.011 |  |  |  |  |  |  |  |  |  |  |
| 1-3 days/week | -0.85 (-0.93, -0.77) | <0.001 |  |  |  |  |  |  |  |  |  |  |
| 4-6 days/week | -0.68 (-0.76, -0.59) | <0.001 |  |  |  |  |  |  |  |  |  |  |
| Daily | -1.02 (-1.10, -0.93) | <0.001 |  |  |  |  |  |  |  |  |  |  |
| Frequency of yogurt (Ref. = Never) | |  |  |  |  |  |  |  |  |  |  |  |
| Less than once per month |  |  | -0.08 (-0.15, -0.01) | 0.018 |  |  |  |  |  |  |  |  |
| 1-3 days/week |  |  | -0.61 (-0.67, -0.54) | <0.001 |  |  |  |  |  |  |  |  |
| 4-6 days/week |  |  | -0.34 (-0.42, -0.26) | <0.001 |  |  |  |  |  |  |  |  |
| Daily |  |  | -0.84 (-0.93, -0.76) | <0.001 |  |  |  |  |  |  |  |  |
| Frequency of milk powder (Ref. = Never) | | |  |  |  |  |  |  |  |  |  |  |
| Less than once per month |  |  |  |  | 0.04 (-0.02, 0.11) | 0.215 |  |  |  |  |  |  |
| 1-3 days/week |  |  |  |  | -0.60 (-0.67, -0.54) | <0.001 |  |  |  |  |  |  |
| 4-6 days/week |  |  |  |  | -0.32 (-0.42, -0.22) | <0.001 |  |  |  |  |  |  |
| Daily |  |  |  |  | -0.92 (-1.01, -0.82) | <0.001 |  |  |  |  |  |  |
| Frequency of cheese/cheese cubes (Ref. = Never) | | |  |  |  |  |  |  |  |  |  |  |
| Less than once per month |  |  |  |  |  |  | 0.05 (-0.00, 0.11) | 0.052 |  |  |  |  |
| 1-3 days/week |  |  |  |  |  |  | -0.55 (-0.62, -0.49) | <0.001 |  |  |  |  |
| 4-6 days/week |  |  |  |  |  |  | -0.35 (-0.46, -0.24) | <0.001 |  |  |  |  |
| Daily |  |  |  |  |  |  | -1.21 (-1.32, -1.11) | <0.001 |  |  |  |  |
| Daily amount of milk, yogurt, and milk powder (200 mL units) |  |  |  |  |  |  |  |  | 0.08 (0.06, 0.11) | <0.001 |  |  |
| Daily amount of cheese/cheese cubes (Ref. = Never) | | |  |  |  |  |  |  |  |  |  |  |
| 1-3 pieces |  |  |  |  |  |  |  |  |  |  | -0.31 (-0.36, -0.26) | <0.001 |
| 4-6 pieces |  |  |  |  |  |  |  |  |  |  | -0.47 (-0.54, -0.39) | <0.001 |
| 7-9 pieces |  |  |  |  |  |  |  |  |  |  | -0.27 (-0.40, -0.13) | <0.001 |
| 10-12 pieces |  |  |  |  |  |  |  |  |  |  | -0.28 (-0.43, -0.12) | <0.001 |
| 13-15 pieces |  |  |  |  |  |  |  |  |  |  | 0.41 (0.15, 0.67) | 0.002 |
| >15 pieces |  |  |  |  |  |  |  |  |  |  | 0.42 (0.15, 0.68) | 0.002 |

Note: Each dairy intake variable was entered separately. Models were adjusted for age, gender, ethnicity, education level, region, body mass index, living on campus, history of smoking, history of drinking, physical inactivity, low fruit or vegetable intake, unhealthy diet, father’s education level, and mother’s education level.

**Table S6. Multiple imputation sensitivity analysis of the associations between dairy intake and depressive and anxiety symptoms**

| Variables | Depressive symptoms | | Anxiety symptoms | |
| --- | --- | --- | --- | --- |
|  | beta (95% CI) | P | beta (95% CI) | P |
| Age | 0.31 (0.27, 0.35) | <0.001 | 0.16 (0.14, 0.18) | <0.001 |
| Gender (Ref. = Girls) | -1.57 (-1.66, -1.49) | <0.001 | -1.09 (-1.13, -1.06) | <0.001 |
| Ethnicity (Ref. = Other) | -0.01 (-0.35, 0.32) | 0.939 | -0.02 (-0.17, 0.13) | 0.814 |
| Education level (Ref. = Junior high school) | -0.41 (-0.57, -0.25) | <0.001 | 0.14 (0.07, 0.21) | <0.001 |
| Region (Ref. = Suburban) | -0.18 (-0.26, -0.09) | <0.001 | -0.08 (-0.12, -0.04) | <0.001 |
| BMI | 0.03 (0.02, 0.04) | <0.001 | 0.01 (0.00, 0.01) | <0.001 |
| Living on campus (Ref. = No) | -0.22 (-0.32, -0.12) | <0.001 | -0.20 (-0.24, -0.15) | <0.001 |
| History of smoking (Ref. = No) | 3.49 (3.23, 3.75) | <0.001 | 1.14 (1.02, 1.25) | <0.001 |
| History of drinking (Ref. = No) | 4.34 (4.20, 4.47) | <0.001 | 1.93 (1.86, 1.99) | <0.001 |
| Physical inactivity (Ref. = No) | 0.35 (0.23, 0.48) | <0.001 | 0.12 (0.06, 0.17) | <0.001 |
| Low fruit or vegetable intake (Ref. = No) | 2.35 (2.26, 2.44) | <0.001 | 0.61 (0.57, 0.65) | <0.001 |
| Unhealthy diet (Ref. = No) | 2.71 (2.58, 2.83) | <0.001 | 1.01 (0.95, 1.07) | <0.001 |
| Father’s education level (Ref. = Junior high school or below) | -0.28 (-0.41, -0.15) | <0.001 | -0.03 (-0.09, 0.04) | 0.422 |
| Mother’s education level (Ref. = Junior high school or below) | -0.4 (-0.51, -0.28) | <0.001 | -0.13 (-0.19, -0.08) | <0.001 |
| Frequency of milk (Ref. = Never) |  |  |  |  |
| Less than once per month | -0.53 (-0.77, -0.29) | <0.001 | -0.19 (-0.28, -0.09) | <0.001 |
| 1-3 days/week | -1.7 (-1.92, -1.49) | <0.001 | -0.66 (-0.75, -0.58) | <0.001 |
| 4-6 days/week | -1.86 (-2.10, -1.62) | <0.001 | -0.62 (-0.71, -0.52) | <0.001 |
| Daily | -2.24 (-2.45, -2.03) | <0.001 | -0.86 (-0.95, -0.77) | <0.001 |
| Frequency of yogurt (Ref. = Never) |  |  |  |  |
| Less than once per month | -0.69 (-0.86, -0.51) | <0.001 | -0.10 (-0.17, -0.03) | 0.008 |
| 1-3 days/week | -1.07 (-1.22, -0.91) | <0.001 | -0.39 (-0.46, -0.32) | <0.001 |
| 4-6 days/week | -0.79 (-0.99, -0.59) | <0.001 | -0.24 (-0.32, -0.15) | <0.001 |
| Daily | -0.86 (-1.08, -0.65) | <0.001 | -0.34 (-0.44, -0.24) | <0.001 |
| Frequency of milk powder (Ref. = Never) |  |  |  |  |
| Less than once per month | 0.14 (-0.01, 0.28) | 0.071 | 0.00 (-0.07, 0.07) | 0.978 |
| 1-3 days/week | 0.21 (0.05, 0.37) | 0.010 | -0.28 (-0.35, -0.2) | <0.001 |
| 4-6 days/week | 0.44 (0.21, 0.67) | <0.001 | -0.17 (-0.28, -0.07) | 0.001 |
| Daily | 0.65 (0.37, 0.93) | <0.001 | -0.21 (-0.34, -0.08) | 0.002 |
| Frequency of cheese/cheese cubes (Ref. = Never) |  |  |  |  |
| Less than once per month | 0.11 (-0.06, 0.28) | 0.190 | 0.11 (0.04, 0.19) | 0.004 |
| 1-3 days/week | 0.28 (0.07, 0.48) | 0.009 | -0.16 (-0.25, -0.08) | <0.001 |
| 4-6 days/week | 0.51 (0.20, 0.81) | 0.001 | -0.21 (-0.35, -0.08) | 0.002 |
| Daily | 0.92 (0.54, 1.30) | <0.001 | -0.60 (-0.76, -0.44) | <0.001 |
| Daily amount of milk, yogurt, and milk powder (200 ml) | 0.10 (0.02, 0.17) | 0.011 | 0.19 (0.16, 0.22) | <0.001 |
| Daily amount of cheese/cheese cubes (Ref. = Never) |  |  |  |  |
| 1-3 pieces | -0.24 (-0.41, -0.07) | 0.004 | -0.05 (-0.13, 0.02) | 0.155 |
| 4-6 pieces | 0.18 (-0.04, 0.41) | 0.114 | -0.11 (-0.21, -0.01) | 0.036 |
| 7-9 pieces | 0.91 (0.60, 1.22) | <0.001 | 0.02 (-0.13, 0.17) | 0.780 |
| 10-12 pieces | 1.08 (0.71, 1.46) | <0.001 | 0.02 (-0.15, 0.19) | 0.820 |
| 13-15 pieces | 1.89 (1.30, 2.47) | <0.001 | 0.31 (0.05, 0.57) | 0.019 |
| >15 pieces | 1.69 (1.05, 2.33) | <0.001 | 0.64 (0.37, 0.91) | <0.001 |

Note: BMI, body mass index; CI, confidence intervals.

**Table S7. Sensitivity analyses of the associations between dairy intake and depressive and anxiety symptoms using alternative outcome specifications**

| Variables | Negative binomial regression | | | | Logistic regression | | | |
| --- | --- | --- | --- | --- | --- | --- | --- | --- |
|  | Depressive symptoms | | Anxiety symptoms | | Depressive symptoms | | Anxiety symptoms | |
|  | IRR (95% CI) | P | IRR (95% CI) | P | AOR (95% CI) | P | AOR (95% CI) | P |
| Age | 1.04 (1.03, 1.04) | <0.001 | 1.08 (1.07, 1.08) | <0.001 | 1.09 (1.07, 1.10) | <0.001 | 1.08 (1.05, 1.10) | <0.001 |
| Gender (Ref. = Girls) | 0.85 (0.85, 0.86) | <0.001 | 0.67 (0.66, 0.68) | <0.001 | 0.59 (0.57, 0.61) | <0.001 | 0.53 (0.50, 0.55) | <0.001 |
| Ethnicity (Ref. = Other) | 0.99 (0.95, 1.03) | 0.511 | 0.99 (0.92, 1.06) | 0.777 | 0.99 (0.87, 1.11) | 0.811 | 0.88 (0.73, 1.05) | 0.163 |
| Education level (Ref. = Junior high school) | 0.96 (0.94, 0.97) | <0.001 | 1.01 (0.98, 1.05) | 0.459 | 0.97 (0.92, 1.03) | 0.286 | 0.85 (0.78, 0.93) | <0.001 |
| Region (Ref. = Suburban) | 0.97 (0.96, 0.98) | <0.001 | 0.98 (0.96, 0.99) | 0.007 | 0.96 (0.94, 0.99) | 0.021 | 1.03 (0.99, 1.08) | 0.177 |
| BMI | 1.00 (1.00, 1.00) | <0.001 | 1.00 (1.00, 1.01) | <0.001 | 1.01 (1.00, 1.01) | <0.001 | 1.01 (1.01, 1.02) | <0.001 |
| Living on campus (Ref. = No) | 0.98 (0.97, 0.99) | 0.004 | 0.94 (0.93, 0.96) | <0.001 | 0.91 (0.88, 0.94) | <0.001 | 0.82 (0.77, 0.86) | <0.001 |
| History of smoking (Ref. = No) | 1.29 (1.25, 1.33) | <0.001 | 1.36 (1.29, 1.43) | <0.001 | 1.74 (1.62, 1.87) | <0.001 | 1.80 (1.64, 1.99) | <0.001 |
| History of drinking (Ref. = No) | 1.47 (1.45, 1.49) | <0.001 | 1.74 (1.69, 1.79) | <0.001 | 2.71 (2.61, 2.82) | <0.001 | 2.50 (2.36, 2.65) | <0.001 |
| Physical inactivity (Ref. = No) | 1.04 (1.02, 1.05) | <0.001 | 1.07 (1.04, 1.09) | <0.001 | 1.04 (1.00, 1.09) | 0.068 | 0.91 (0.85, 0.97) | 0.007 |
| Low fruit or vegetable intake (Ref. = No) | 1.27 (1.26, 1.29) | <0.001 | 1.24 (1.22, 1.26) | <0.001 | 1.64 (1.59, 1.69) | <0.001 | 1.49 (1.42, 1.56) | <0.001 |
| Unhealthy diet (Ref. = No) | 1.29 (1.27, 1.31) | <0.001 | 1.40 (1.37, 1.44) | <0.001 | 1.83 (1.76, 1.91) | <0.001 | 1.90 (1.79, 2.01) | <0.001 |
| Father’s education level (Ref. = Junior high school or below) | 0.97 (0.95, 0.98) | <0.001 | 0.99 (0.97, 1.01) | 0.261 | 0.97 (0.94, 1.01) | 0.168 | 1.02 (0.96, 1.08) | 0.545 |
| Mother’s education level (Ref. = Junior high school or below) | 0.95 (0.94, 0.97) | <0.001 | 0.95 (0.93, 0.97) | <0.001 | 0.93 (0.90, 0.97) | <0.001 | 0.98 (0.93, 1.04) | 0.514 |
| Frequency of milk (Ref. = Never) |  |  |  |  |  |  |  |  |
| Less than once per month | 0.96 (0.94, 0.98) | 0.001 | 0.94 (0.90, 0.98) | 0.003 | 0.94 (0.88, 1.00) | 0.053 | 0.75 (0.68, 0.82) | <0.001 |
| 1-3 days/week | 0.85 (0.84, 0.87) | <0.001 | 0.8 (0.78, 0.83) | <0.001 | 0.68 (0.64, 0.72) | <0.001 | 0.56 (0.51, 0.60) | <0.001 |
| 4-6 days/week | 0.84 (0.82, 0.85) | <0.001 | 0.82 (0.79, 0.85) | <0.001 | 0.71 (0.67, 0.75) | <0.001 | 0.60 (0.55, 0.66) | <0.001 |
| Daily | 0.80 (0.78, 0.82) | <0.001 | 0.73 (0.70, 0.76) | <0.001 | 0.61 (0.57, 0.65) | <0.001 | 0.52 (0.47, 0.57) | <0.001 |
| Frequency of yogurt (Ref. = Never) |  |  |  |  |  |  |  |  |
| Less than once per month | 0.93 (0.91, 0.94) | <0.001 | 0.96 (0.94, 0.99) | 0.015 | 0.86 (0.82, 0.90) | <0.001 | 0.75 (0.69, 0.80) | <0.001 |
| 1-3 days/week | 0.89 (0.88, 0.91) | <0.001 | 0.85 (0.82, 0.87) | <0.001 | 0.70 (0.67, 0.74) | <0.001 | 0.63 (0.59, 0.68) | <0.001 |
| 4-6 days/week | 0.92 (0.90, 0.93) | <0.001 | 0.9 (0.87, 0.94) | <0.001 | 0.82 (0.78, 0.88) | <0.001 | 0.72 (0.66, 0.79) | <0.001 |
| Daily | 0.92 (0.90, 0.94) | <0.001 | 0.87 (0.83, 0.90) | <0.001 | 0.78 (0.73, 0.84) | <0.001 | 0.76 (0.69, 0.85) | <0.001 |
| Frequency of milk powder (Ref. = Never) |  |  |  |  |  |  |  |  |
| Less than once per month | 1.01 (1.00, 1.03) | 0.086 | 1.01 (0.98, 1.04) | 0.385 | 0.99 (0.94, 1.05) | 0.802 | 1.01 (0.93, 1.09) | 0.869 |
| 1-3 days/week | 1.03 (1.01, 1.05) | 0.002 | 0.89 (0.86, 0.92) | <0.001 | 0.93 (0.87, 0.98) | 0.011 | 0.85 (0.77, 0.94) | 0.001 |
| 4-6 days/week | 1.04 (1.02, 1.07) | 0.001 | 0.92 (0.88, 0.97) | 0.002 | 1.07 (0.98, 1.16) | 0.126 | 1.08 (0.95, 1.23) | 0.223 |
| Daily | 1.08 (1.05, 1.11) | <0.001 | 0.90 (0.86, 0.95) | <0.001 | 0.99 (0.90, 1.09) | 0.899 | 1.13 (0.98, 1.30) | 0.091 |
| Frequency of cheese/cheese cubes (Ref. = Never) |  |  |  |  |  |  |  |  |
| Less than once per month | 1.01 (0.99, 1.03) | 0.164 | 1.06 (1.03, 1.10) | <0.001 | 1.05 (0.99, 1.11) | 0.125 | 0.99 (0.90, 1.09) | 0.824 |
| 1-3 days/week | 1.03 (1.01, 1.06) | 0.006 | 0.95 (0.91, 0.99) | 0.009 | 0.99 (0.92, 1.06) | 0.758 | 0.95 (0.85, 1.07) | 0.416 |
| 4-6 days/week | 1.07 (1.03, 1.11) | <0.001 | 0.96 (0.90, 1.01) | 0.135 | 1.06 (0.96, 1.18) | 0.241 | 1.00 (0.85, 1.17) | 0.989 |
| Daily | 1.12 (1.08, 1.16) | <0.001 | 0.71 (0.67, 0.77) | <0.001 | 0.72 (0.63, 0.82) | <0.001 | 0.92 (0.76, 1.10) | 0.358 |
| Daily amount of milk, yogurt, and milk powder (200 ml) | 1.01 (1.01, 1.02) | <0.001 | 1.09 (1.07, 1.10) | <0.001 | 1.10 (1.08, 1.12) | <0.001 | 1.10 (1.06, 1.13) | <0.001 |
| Daily amount of cheese/cheese cubes (Ref. = Never) |  |  |  |  |  |  |  |  |
| 1-3 pieces | 0.97 (0.95, 0.98) | <0.001 | 0.96 (0.93, 1.00) | 0.025 | 0.91 (0.85, 0.96) | <0.001 | 0.97 (0.89, 1.07) | 0.563 |
| 4-6 pieces | 1.02 (0.99, 1.04) | 0.145 | 0.95 (0.91, 0.99) | 0.027 | 0.94 (0.87, 1.02) | 0.128 | 0.91 (0.80, 1.04) | 0.155 |
| 7-9 pieces | 1.11 (1.07, 1.15) | <0.001 | 1.01 (0.95, 1.07) | 0.799 | 1.09 (0.98, 1.21) | 0.126 | 1.33 (1.13, 1.57) | <0.001 |
| 10-12 pieces | 1.11 (1.06, 1.15) | <0.001 | 0.99 (0.91, 1.06) | 0.691 | 1.17 (1.03, 1.32) | 0.013 | 1.33 (1.10, 1.60) | 0.003 |
| 13-15 pieces | 1.23 (1.15, 1.31) | <0.001 | 1.19 (1.06, 1.33) | 0.003 | 1.25 (1.05, 1.50) | 0.014 | 1.90 (1.49, 2.42) | <0.001 |
| >15 pieces | 1.17 (1.10, 1.25) | <0.001 | 1.35 (1.20, 1.52) | <0.001 | 1.49 (1.23, 1.79) | <0.001 | 2.09 (1.65, 2.65) | <0.001 |

Note: Negative binomial regression results are presented as incidence rate ratios (IRRs) with 95% CIs, whereas logistic regression results are presented as odds ratios (ORs) with 95% CIs. All six dairy intake variables were included simultaneously, and models were adjusted for the same covariates as the primary analyses. BMI, body mass index; CI, confidence intervals; IRR, incidence rate ratio; AOR, adjusted odds ratios.

**Table S8. Interaction analyses of dairy intake variables with gender and region for depressive and anxiety symptoms**

| Outcome | Model | Modifier | Dairy intake | Interaction term | Beta (95%CI) | P value | P for interaction | FDR P for interaction |
| --- | --- | --- | --- | --- | --- | --- | --- | --- |
| Depressive symptoms | Model 1 | Gender (Ref. = Girls) | Frequency of milk (Ref. = Never) | Less than once per month × Boys | -0.17 (-0.59, 0.25) | 0.436 | <0.001 | <0.001 |
| Depressive symptoms | Model 1 | Gender (Ref. = Girls) | Frequency of milk (Ref. = Never) | 1-3 days/week × Boys | 0.50 (0.14, 0.86) | 0.007 |  |  |
| Depressive symptoms | Model 1 | Gender (Ref. = Girls) | Frequency of milk (Ref. = Never) | 4-6 days/week × Boys | 0.63 (0.25, 1.02) | 0.001 |  |  |
| Depressive symptoms | Model 1 | Gender (Ref. = Girls) | Frequency of milk (Ref. = Never) | Daily × Boys | 0.35 (-0.02, 0.72) | 0.062 |  |  |
| Depressive symptoms | Model 2 | Gender (Ref. = Girls) | Frequency of yogurt (Ref. = Never) | Less than once per month × Boys | 0.38 (0.08, 0.69) | 0.014 | <0.001 | <0.001 |
| Depressive symptoms | Model 2 | Gender (Ref. = Girls) | Frequency of yogurt (Ref. = Never) | 1-3 days/week × Boys | 0.85 (0.57, 1.13) | <0.001 |  |  |
| Depressive symptoms | Model 2 | Gender (Ref. = Girls) | Frequency of yogurt (Ref. = Never) | 4-6 days/week × Boys | 0.56 (0.22, 0.91) | 0.001 |  |  |
| Depressive symptoms | Model 2 | Gender (Ref. = Girls) | Frequency of yogurt (Ref. = Never) | Daily × Boys | 0.78 (0.41, 1.15) | <0.001 |  |  |
| Depressive symptoms | Model 3 | Gender (Ref. = Girls) | Frequency of milk powder (Ref. = Never) | Less than once per month × Boys | 0.54 (0.24, 0.83) | <0.001 | <0.001 | <0.001 |
| Depressive symptoms | Model 3 | Gender (Ref. = Girls) | Frequency of milk powder (Ref. = Never) | 1-3 days/week × Boys | 0.95 (0.67, 1.23) | <0.001 |  |  |
| Depressive symptoms | Model 3 | Gender (Ref. = Girls) | Frequency of milk powder (Ref. = Never) | 4-6 days/week × Boys | 1.52 (1.08, 1.95) | <0.001 |  |  |
| Depressive symptoms | Model 3 | Gender (Ref. = Girls) | Frequency of milk powder (Ref. = Never) | Daily × Boys | 0.81 (0.40, 1.22) | <0.001 |  |  |
| Depressive symptoms | Model 4 | Gender (Ref. = Girls) | Frequency of cheese/cheese cubes (Ref. = Never) | Less than once per month × Boys | 0.10 (-0.14, 0.34) | 0.432 | <0.001 | <0.001 |
| Depressive symptoms | Model 4 | Gender (Ref. = Girls) | Frequency of cheese/cheese cubes (Ref. = Never) | 1-3 days/week × Boys | 0.82 (0.54, 1.10) | <0.001 |  |  |
| Depressive symptoms | Model 4 | Gender (Ref. = Girls) | Frequency of cheese/cheese cubes (Ref. = Never) | 4-6 days/week × Boys | 1.39 (0.92, 1.86) | <0.001 |  |  |
| Depressive symptoms | Model 4 | Gender (Ref. = Girls) | Frequency of cheese/cheese cubes (Ref. = Never) | Daily × Boys | 1.07 (0.60, 1.55) | <0.001 |  |  |
| Depressive symptoms | Model 5 | Gender (Ref. = Girls) | Daily amount of milk, yogurt, and milk powder (200 mL units) | Daily amount of milk, yogurt, and milk powder (200 mL units) × Boys | -0.20 (-0.32, -0.07) | 0.002 | 0.002 | 0.002 |
| Depressive symptoms | Model 6 | Gender (Ref. = Girls) | Daily amount of cheese/cheese cubes (Ref. = Never) | 1-3 pieces × Boys | 0.34 (0.12, 0.56) | 0.003 | <0.001 | <0.001 |
| Depressive symptoms | Model 6 | Gender (Ref. = Girls) | Daily amount of cheese/cheese cubes (Ref. = Never) | 4-6 pieces × Boys | 0.40 (0.06, 0.75) | 0.023 |  |  |
| Depressive symptoms | Model 6 | Gender (Ref. = Girls) | Daily amount of cheese/cheese cubes (Ref. = Never) | 7-9 pieces × Boys | 1.03 (0.46, 1.60) | <0.001 |  |  |
| Depressive symptoms | Model 6 | Gender (Ref. = Girls) | Daily amount of cheese/cheese cubes (Ref. = Never) | 10-12 pieces × Boys | 2.18 (1.50, 2.86) | <0.001 |  |  |
| Depressive symptoms | Model 6 | Gender (Ref. = Girls) | Daily amount of cheese/cheese cubes (Ref. = Never) | 13-15 pieces × Boys | 1.99 (0.78, 3.20) | 0.001 |  |  |
| Depressive symptoms | Model 6 | Gender (Ref. = Girls) | Daily amount of cheese/cheese cubes (Ref. = Never) | >15 pieces × Boys | -0.35 (-1.58, 0.88) | 0.576 |  |  |
| Depressive symptoms | Model 7 | Region (Ref. = Suburban) | Frequency of milk (Ref. = Never) | Less than once per month × Urban | 0.30 (-0.12, 0.72) | 0.163 | <0.001 | 0.001 |
| Depressive symptoms | Model 7 | Region (Ref. = Suburban) | Frequency of milk (Ref. = Never) | 1-3 days/week × Urban | 0.40 (0.05, 0.76) | 0.027 |  |  |
| Depressive symptoms | Model 7 | Region (Ref. = Suburban) | Frequency of milk (Ref. = Never) | 4-6 days/week × Urban | 0.68 (0.30, 1.06) | <0.001 |  |  |
| Depressive symptoms | Model 7 | Region (Ref. = Suburban) | Frequency of milk (Ref. = Never) | Daily × Urban | 0.70 (0.34, 1.07) | <0.001 |  |  |
| Depressive symptoms | Model 8 | Region (Ref. = Suburban) | Frequency of yogurt (Ref. = Never) | Less than once per month × Urban | -0.14 (-0.43, 0.16) | 0.371 | 0.629 | 0.629 |
| Depressive symptoms | Model 8 | Region (Ref. = Suburban) | Frequency of yogurt (Ref. = Never) | 1-3 days/week × Urban | -0.19 (-0.47, 0.08) | 0.174 |  |  |
| Depressive symptoms | Model 8 | Region (Ref. = Suburban) | Frequency of yogurt (Ref. = Never) | 4-6 days/week × Urban | -0.03 (-0.37, 0.31) | 0.875 |  |  |
| Depressive symptoms | Model 8 | Region (Ref. = Suburban) | Frequency of yogurt (Ref. = Never) | Daily × Urban | -0.15 (-0.51, 0.21) | 0.423 |  |  |
| Depressive symptoms | Model 9 | Region (Ref. = Suburban) | Frequency of milk powder (Ref. = Never) | Less than once per month × Urban | 0.34 (0.05, 0.64) | 0.022 | 0.014 | 0.020 |
| Depressive symptoms | Model 9 | Region (Ref. = Suburban) | Frequency of milk powder (Ref. = Never) | 1-3 days/week × Urban | -0.25 (-0.53, 0.03) | 0.079 |  |  |
| Depressive symptoms | Model 9 | Region (Ref. = Suburban) | Frequency of milk powder (Ref. = Never) | 4-6 days/week × Urban | -0.35 (-0.78, 0.08) | 0.115 |  |  |
| Depressive symptoms | Model 9 | Region (Ref. = Suburban) | Frequency of milk powder (Ref. = Never) | Daily × Urban | -0.14 (-0.53, 0.26) | 0.491 |  |  |
| Depressive symptoms | Model 10 | Region (Ref. = Suburban) | Frequency of cheese/cheese cubes (Ref. = Never) | Less than once per month × Urban | 0.35 (0.11, 0.59) | 0.004 | 0.008 | 0.016 |
| Depressive symptoms | Model 10 | Region (Ref. = Suburban) | Frequency of cheese/cheese cubes (Ref. = Never) | 1-3 days/week × Urban | -0.02 (-0.30, 0.26) | 0.885 |  |  |
| Depressive symptoms | Model 10 | Region (Ref. = Suburban) | Frequency of cheese/cheese cubes (Ref. = Never) | 4-6 days/week × Urban | -0.16 (-0.62, 0.31) | 0.505 |  |  |
| Depressive symptoms | Model 10 | Region (Ref. = Suburban) | Frequency of cheese/cheese cubes (Ref. = Never) | Daily × Urban | -0.41 (-0.88, 0.05) | 0.079 |  |  |
| Depressive symptoms | Model 11 | Region (Ref. = Suburban) | Daily amount of milk, yogurt, and milk powder (200 mL units) | Daily amount of milk, yogurt, and milk powder (200 mL units) × Urban | 0.20 (0.08, 0.33) | 0.001 | 0.001 | 0.004 |
| Depressive symptoms | Model 12 | Region (Ref. = Suburban) | Daily amount of cheese/cheese cubes (Ref. = Never) | 1-3 pieces × Urban | 0.17 (-0.04, 0.39) | 0.117 | 0.018 | 0.022 |
| Depressive symptoms | Model 12 | Region (Ref. = Suburban) | Daily amount of cheese/cheese cubes (Ref. = Never) | 4-6 pieces × Urban | 0.06 (-0.29, 0.41) | 0.731 |  |  |
| Depressive symptoms | Model 12 | Region (Ref. = Suburban) | Daily amount of cheese/cheese cubes (Ref. = Never) | 7-9 pieces × Urban | 0.21 (-0.36, 0.78) | 0.465 |  |  |
| Depressive symptoms | Model 12 | Region (Ref. = Suburban) | Daily amount of cheese/cheese cubes (Ref. = Never) | 10-12 pieces × Urban | -0.50 (-1.18, 0.18) | 0.153 |  |  |
| Depressive symptoms | Model 12 | Region (Ref. = Suburban) | Daily amount of cheese/cheese cubes (Ref. = Never) | 13-15 pieces × Urban | -1.49 (-2.63, -0.35) | 0.010 |  |  |
| Depressive symptoms | Model 12 | Region (Ref. = Suburban) | Daily amount of cheese/cheese cubes (Ref. = Never) | >15 pieces × Urban | 1.07 (-0.10, 2.23) | 0.073 |  |  |
| Anxiety symptoms | Model 13 | Gender (Ref. = Girls) | Frequency of milk (Ref. = Never) | Less than once per month × Boys | -0.02 (-0.21, 0.17) | 0.853 | <0.001 | <0.001 |
| Anxiety symptoms | Model 13 | Gender (Ref. = Girls) | Frequency of milk (Ref. = Never) | 1-3 days/week × Boys | 0.24 (0.07, 0.40) | 0.005 |  |  |
| Anxiety symptoms | Model 13 | Gender (Ref. = Girls) | Frequency of milk (Ref. = Never) | 4-6 days/week × Boys | 0.32 (0.14, 0.49) | <0.001 |  |  |
| Anxiety symptoms | Model 13 | Gender (Ref. = Girls) | Frequency of milk (Ref. = Never) | Daily × Boys | 0.25 (0.08, 0.42) | 0.004 |  |  |
| Anxiety symptoms | Model 14 | Gender (Ref. = Girls) | Frequency of yogurt (Ref. = Never) | Less than once per month × Boys | 0.14 (0.00, 0.28) | 0.047 | <0.001 | <0.001 |
| Anxiety symptoms | Model 14 | Gender (Ref. = Girls) | Frequency of yogurt (Ref. = Never) | 1-3 days/week × Boys | 0.30 (0.17, 0.43) | <0.001 |  |  |
| Anxiety symptoms | Model 14 | Gender (Ref. = Girls) | Frequency of yogurt (Ref. = Never) | 4-6 days/week × Boys | 0.11 (-0.05, 0.27) | 0.185 |  |  |
| Anxiety symptoms | Model 14 | Gender (Ref. = Girls) | Frequency of yogurt (Ref. = Never) | Daily × Boys | 0.12 (-0.05, 0.29) | 0.157 |  |  |
| Anxiety symptoms | Model 15 | Gender (Ref. = Girls) | Frequency of milk powder (Ref. = Never) | Less than once per month × Boys | 0.26 (0.13, 0.40) | <0.001 | <0.001 | <0.001 |
| Anxiety symptoms | Model 15 | Gender (Ref. = Girls) | Frequency of milk powder (Ref. = Never) | 1-3 days/week × Boys | 0.50 (0.37, 0.63) | <0.001 |  |  |
| Anxiety symptoms | Model 15 | Gender (Ref. = Girls) | Frequency of milk powder (Ref. = Never) | 4-6 days/week × Boys | 0.55 (0.35, 0.75) | <0.001 |  |  |
| Anxiety symptoms | Model 15 | Gender (Ref. = Girls) | Frequency of milk powder (Ref. = Never) | Daily × Boys | 0.31 (0.12, 0.49) | 0.001 |  |  |
| Anxiety symptoms | Model 16 | Gender (Ref. = Girls) | Frequency of cheese/cheese cubes (Ref. = Never) | Less than once per month × Boys | 0.03 (-0.08, 0.14) | 0.582 | <0.001 | <0.001 |
| Anxiety symptoms | Model 16 | Gender (Ref. = Girls) | Frequency of cheese/cheese cubes (Ref. = Never) | 1-3 days/week × Boys | 0.33 (0.20, 0.46) | <0.001 |  |  |
| Anxiety symptoms | Model 16 | Gender (Ref. = Girls) | Frequency of cheese/cheese cubes (Ref. = Never) | 4-6 days/week × Boys | 0.42 (0.20, 0.63) | <0.001 |  |  |
| Anxiety symptoms | Model 16 | Gender (Ref. = Girls) | Frequency of cheese/cheese cubes (Ref. = Never) | Daily × Boys | 0.35 (0.13, 0.56) | 0.002 |  |  |
| Anxiety symptoms | Model 17 | Gender (Ref. = Girls) | Daily amount of milk, yogurt, and milk powder (200 mL units) | Daily amount of milk, yogurt, and milk powder (200 mL units) × Boys | -0.04 (-0.10, 0.02) | 0.171 | 0.171 | 0.171 |
| Anxiety symptoms | Model 18 | Gender (Ref. = Girls) | Daily amount of cheese/cheese cubes (Ref. = Never) | 1-3 pieces × Boys | 0.07 (-0.03, 0.17) | 0.166 | <0.001 | <0.001 |
| Anxiety symptoms | Model 18 | Gender (Ref. = Girls) | Daily amount of cheese/cheese cubes (Ref. = Never) | 4-6 pieces × Boys | 0.15 (-0.01, 0.31) | 0.067 |  |  |
| Anxiety symptoms | Model 18 | Gender (Ref. = Girls) | Daily amount of cheese/cheese cubes (Ref. = Never) | 7-9 pieces × Boys | 0.21 (-0.05, 0.47) | 0.121 |  |  |
| Anxiety symptoms | Model 18 | Gender (Ref. = Girls) | Daily amount of cheese/cheese cubes (Ref. = Never) | 10-12 pieces × Boys | 0.86 (0.55, 1.17) | <0.001 |  |  |
| Anxiety symptoms | Model 18 | Gender (Ref. = Girls) | Daily amount of cheese/cheese cubes (Ref. = Never) | 13-15 pieces × Boys | 0.29 (-0.27, 0.84) | 0.311 |  |  |
| Anxiety symptoms | Model 18 | Gender (Ref. = Girls) | Daily amount of cheese/cheese cubes (Ref. = Never) | >15 pieces × Boys | -0.52 (-1.08, 0.04) | 0.070 |  |  |
| Anxiety symptoms | Model 19 | Region (Ref. = Suburban) | Frequency of milk (Ref. = Never) | Less than once per month × Urban | 0.03 (-0.16, 0.22) | 0.740 | <0.001 | <0.001 |
| Anxiety symptoms | Model 19 | Region (Ref. = Suburban) | Frequency of milk (Ref. = Never) | 1-3 days/week × Urban | 0.14 (-0.02, 0.30) | 0.090 |  |  |
| Anxiety symptoms | Model 19 | Region (Ref. = Suburban) | Frequency of milk (Ref. = Never) | 4-6 days/week × Urban | 0.29 (0.11, 0.46) | 0.001 |  |  |
| Anxiety symptoms | Model 19 | Region (Ref. = Suburban) | Frequency of milk (Ref. = Never) | Daily × Urban | 0.30 (0.13, 0.47) | <0.001 |  |  |
| Anxiety symptoms | Model 20 | Region (Ref. = Suburban) | Frequency of yogurt (Ref. = Never) | Less than once per month × Urban | -0.12 (-0.25, 0.02) | 0.098 | 0.389 | 0.389 |
| Anxiety symptoms | Model 20 | Region (Ref. = Suburban) | Frequency of yogurt (Ref. = Never) | 1-3 days/week × Urban | -0.05 (-0.18, 0.07) | 0.394 |  |  |
| Anxiety symptoms | Model 20 | Region (Ref. = Suburban) | Frequency of yogurt (Ref. = Never) | 4-6 days/week × Urban | -0.09 (-0.24, 0.07) | 0.266 |  |  |
| Anxiety symptoms | Model 20 | Region (Ref. = Suburban) | Frequency of yogurt (Ref. = Never) | Daily × Urban | 0.00 (-0.16, 0.17) | 0.983 |  |  |
| Anxiety symptoms | Model 21 | Region (Ref. = Suburban) | Frequency of milk powder (Ref. = Never) | Less than once per month × Urban | 0.05 (-0.08, 0.19) | 0.451 | 0.220 | 0.264 |
| Anxiety symptoms | Model 21 | Region (Ref. = Suburban) | Frequency of milk powder (Ref. = Never) | 1-3 days/week × Urban | 0.15 (0.02, 0.28) | 0.021 |  |  |
| Anxiety symptoms | Model 21 | Region (Ref. = Suburban) | Frequency of milk powder (Ref. = Never) | 4-6 days/week × Urban | -0.02 (-0.22, 0.18) | 0.862 |  |  |
| Anxiety symptoms | Model 21 | Region (Ref. = Suburban) | Frequency of milk powder (Ref. = Never) | Daily × Urban | 0.02 (-0.16, 0.20) | 0.809 |  |  |
| Anxiety symptoms | Model 22 | Region (Ref. = Suburban) | Frequency of cheese/cheese cubes (Ref. = Never) | Less than once per month × Urban | 0.16 (0.05, 0.27) | 0.004 | <0.001 | <0.001 |
| Anxiety symptoms | Model 22 | Region (Ref. = Suburban) | Frequency of cheese/cheese cubes (Ref. = Never) | 1-3 days/week × Urban | 0.30 (0.17, 0.42) | <0.001 |  |  |
| Anxiety symptoms | Model 22 | Region (Ref. = Suburban) | Frequency of cheese/cheese cubes (Ref. = Never) | 4-6 days/week × Urban | 0.02 (-0.19, 0.23) | 0.864 |  |  |
| Anxiety symptoms | Model 22 | Region (Ref. = Suburban) | Frequency of cheese/cheese cubes (Ref. = Never) | Daily × Urban | 0.03 (-0.18, 0.24) | 0.770 |  |  |
| Anxiety symptoms | Model 23 | Region (Ref. = Suburban) | Daily amount of milk, yogurt, and milk powder (200 mL units) | Daily amount of milk, yogurt, and milk powder (200 mL units) × Urban | 0.06 (0.01, 0.12) | 0.032 | 0.032 | 0.049 |
| Anxiety symptoms | Model 24 | Region (Ref. = Suburban) | Daily amount of cheese/cheese cubes (Ref. = Never) | 1-3 pieces × Urban | 0.24 (0.15, 0.34) | <0.001 | <0.001 | <0.001 |
| Anxiety symptoms | Model 24 | Region (Ref. = Suburban) | Daily amount of cheese/cheese cubes (Ref. = Never) | 4-6 pieces × Urban | 0.30 (0.14, 0.46) | <0.001 |  |  |
| Anxiety symptoms | Model 24 | Region (Ref. = Suburban) | Daily amount of cheese/cheese cubes (Ref. = Never) | 7-9 pieces × Urban | 0.22 (-0.04, 0.48) | 0.095 |  |  |
| Anxiety symptoms | Model 24 | Region (Ref. = Suburban) | Daily amount of cheese/cheese cubes (Ref. = Never) | 10-12 pieces × Urban | -0.54 (-0.86, -0.23) | <0.001 |  |  |
| Anxiety symptoms | Model 24 | Region (Ref. = Suburban) | Daily amount of cheese/cheese cubes (Ref. = Never) | 13-15 pieces × Urban | -0.19 (-0.71, 0.33) | 0.472 |  |  |
| Anxiety symptoms | Model 24 | Region (Ref. = Suburban) | Daily amount of cheese/cheese cubes (Ref. = Never) | >15 pieces × Urban | 0.12 (-0.42, 0.65) | 0.671 |  |  |

Note: BMI, body mass index; CI, confidence intervals; FDR, false discovery rate. Each model included all six dairy intake variables and covariates simultaneously, with one interaction term added at a time. The P for interaction was obtained from a joint test of interaction terms for categorical dairy variables and from the product term for continuous dairy amount. Benjamini-Hochberg FDR correction was applied separately to the six overall interaction tests within each outcome-modifier combination.

**Table S9. Subgroup-specific associations between dairy intake variables and depressive and anxiety symptoms stratified by gender**

| Variable | Girls | | Boys | | P for interaction | FDR P for interaction |
| --- | --- | --- | --- | --- | --- | --- |
|  | Beta (95%CI) | P | Beta (95%CI) | P |  |  |
| **Depressive symptoms** |  |  |  |  |  |  |
| Frequency of milk (Ref. = Never) |  |  |  |  | <0.001 | <0.001 |
| Less than once per month | -0.15 (-0.28, -0.01) | 0.038 | -0.22 (-0.36, -0.07) | 0.003 |  |  |
| 1-3 days/week | -0.70 (-0.82, -0.58) | <0.001 | -0.66 (-0.78, -0.53) | <0.001 |  |  |
| 4-6 days/week | -0.68 (-0.82, -0.55) | <0.001 | -0.54 (-0.67, -0.40) | <0.001 |  |  |
| Daily | -0.91 (-1.04, -0.78) | <0.001 | -0.81 (-0.94, -0.68) | <0.001 |  |  |
| Frequency of yogurt (Ref. = Never) |  |  |  |  | <0.001 | <0.001 |
| Less than once per month | -0.17 (-0.29, -0.06) | 0.004 | -0.06 (-0.15, 0.03) | 0.189 |  |  |
| 1-3 days/week | -0.51 (-0.62, -0.40) | <0.001 | -0.37 (-0.45, -0.28) | <0.001 |  |  |
| 4-6 days/week | -0.22 (-0.35, -0.09) | 0.001 | -0.32 (-0.43, -0.20) | <0.001 |  |  |
| Daily | -0.34 (-0.49, -0.18) | <0.001 | -0.43 (-0.55, -0.31) | <0.001 |  |  |
| Frequency of milk powder (Ref. = Never) |  |  |  |  | <0.001 | <0.001 |
| Less than once per month | -0.11 (-0.21, -0.01) | 0.037 | 0.16 (0.06, 0.26) | 0.001 |  |  |
| 1-3 days/week | -0.47 (-0.58, -0.36) | <0.001 | -0.10 (-0.20, 0.01) | 0.068 |  |  |
| 4-6 days/week | -0.40 (-0.58, -0.23) | <0.001 | 0.00 (-0.15, 0.16) | 0.950 |  |  |
| Daily | -0.32 (-0.52, -0.13) | 0.001 | -0.12 (-0.27, 0.04) | 0.147 |  |  |
| Frequency of cheese/cheese cubes (Ref. = Never) |  |  |  |  | <0.001 | <0.001 |
| Less than once per month | 0.07 (-0.05, 0.19) | 0.277 | 0.14 (0.03, 0.25) | 0.014 |  |  |
| 1-3 days/week | -0.30 (-0.44, -0.15) | <0.001 | -0.08 (-0.21, 0.05) | 0.225 |  |  |
| 4-6 days/week | -0.38 (-0.59, -0.16) | <0.001 | -0.12 (-0.31, 0.06) | 0.193 |  |  |
| Daily | -0.93 (-1.19, -0.67) | <0.001 | -0.50 (-0.71, -0.30) | <0.001 |  |  |
| Daily amount of milk, yogurt, and milk powder (200 mL units) | 0.25 (0.20, 0.30) | <0.001 | 0.15 (0.11, 0.19) | <0.001 | 0.171 | 0.171 |
| Daily amount of cheese/cheese cubes (Ref. = Never) |  |  |  |  | <0.001 | <0.001 |
| 1-3 pieces | 0.07 (-0.05, 0.19) | 0.247 | -0.15 (-0.26, -0.05) | 0.005 |  |  |
| 4-6 pieces | 0.01 (-0.15, 0.17) | 0.932 | -0.23 (-0.36, -0.09) | 0.001 |  |  |
| 7-9 pieces | 0.07 (-0.16, 0.30) | 0.566 | -0.06 (-0.25, 0.13) | 0.550 |  |  |
| 10-12 pieces | -0.32 (-0.60, -0.04) | 0.027 | 0.18 (-0.04, 0.40) | 0.104 |  |  |
| 13-15 pieces | 0.27 (-0.22, 0.75) | 0.281 | 0.34 (0.02, 0.66) | 0.036 |  |  |
| >15 pieces | 1.13 (0.64, 1.61) | <0.001 | 0.48 (0.15, 0.82) | 0.004 |  |  |
| **Anxiety symptoms** |  |  |  |  |  |  |
| Frequency of milk (Ref. = Never) |  |  |  |  | <0.001 | <0.001 |
| Less than once per month | -0.15 (-0.28, -0.01) | 0.038 | -0.22 (-0.36, -0.07) | 0.003 |  |  |
| 1-3 days/week | -0.70 (-0.82, -0.58) | <0.001 | -0.66 (-0.78, -0.53) | <0.001 |  |  |
| 4-6 days/week | -0.68 (-0.82, -0.55) | <0.001 | -0.54 (-0.67, -0.40) | <0.001 |  |  |
| Daily | -0.91 (-1.04, -0.78) | <0.001 | -0.81 (-0.94, -0.68) | <0.001 |  |  |
| Frequency of yogurt (Ref. = Never) |  |  |  |  | <0.001 | <0.001 |
| Less than once per month | -0.17 (-0.29, -0.06) | 0.004 | -0.06 (-0.15, 0.03) | 0.189 |  |  |
| 1-3 days/week | -0.51 (-0.62, -0.40) | <0.001 | -0.37 (-0.45, -0.28) | <0.001 |  |  |
| 4-6 days/week | -0.22 (-0.35, -0.09) | 0.001 | -0.32 (-0.43, -0.20) | <0.001 |  |  |
| Daily | -0.34 (-0.49, -0.18) | <0.001 | -0.43 (-0.55, -0.31) | <0.001 |  |  |
| Frequency of milk powder (Ref. = Never) |  |  |  |  | <0.001 | <0.001 |
| Less than once per month | -0.11 (-0.21, -0.01) | 0.037 | 0.16 (0.06, 0.26) | 0.001 |  |  |
| 1-3 days/week | -0.47 (-0.58, -0.36) | <0.001 | -0.10 (-0.20, 0.01) | 0.068 |  |  |
| 4-6 days/week | -0.40 (-0.58, -0.23) | <0.001 | 0.00 (-0.15, 0.16) | 0.950 |  |  |
| Daily | -0.32 (-0.52, -0.13) | 0.001 | -0.12 (-0.27, 0.04) | 0.147 |  |  |
| Frequency of cheese/cheese cubes (Ref. = Never) |  |  |  |  | <0.001 | <0.001 |
| Less than once per month | 0.07 (-0.05, 0.19) | 0.277 | 0.14 (0.03, 0.25) | 0.014 |  |  |
| 1-3 days/week | -0.30 (-0.44, -0.15) | <0.001 | -0.08 (-0.21, 0.05) | 0.225 |  |  |
| 4-6 days/week | -0.38 (-0.59, -0.16) | <0.001 | -0.12 (-0.31, 0.06) | 0.193 |  |  |
| Daily | -0.93 (-1.19, -0.67) | <0.001 | -0.50 (-0.71, -0.30) | <0.001 |  |  |
| Daily amount of milk, yogurt, and milk powder (200 mL units) | 0.25 (0.20, 0.30) | <0.001 | 0.15 (0.11, 0.19) | <0.001 | 0.171 | 0.171 |
| Daily amount of cheese/cheese cubes (Ref. = Never) |  |  |  |  | <0.001 | <0.001 |
| 1-3 pieces | 0.07 (-0.05, 0.19) | 0.247 | -0.15 (-0.26, -0.05) | 0.005 |  |  |
| 4-6 pieces | 0.01 (-0.15, 0.17) | 0.932 | -0.23 (-0.36, -0.09) | 0.001 |  |  |
| 7-9 pieces | 0.07 (-0.16, 0.30) | 0.566 | -0.06 (-0.25, 0.13) | 0.550 |  |  |
| 10-12 pieces | -0.32 (-0.60, -0.04) | 0.027 | 0.18 (-0.04, 0.40) | 0.104 |  |  |
| 13-15 pieces | 0.27 (-0.22, 0.75) | 0.281 | 0.34 (0.02, 0.66) | 0.036 |  |  |
| >15 pieces | 1.13 (0.64, 1.61) | <0.001 | 0.48 (0.15, 0.82) | 0.004 |  |  |

Note: BMI, body mass index; CI, confidence intervals; FDR, false discovery rate. Each model included all six dairy intake variables and covariates simultaneously. The P for interaction was obtained from a joint test of interaction terms for categorical dairy variables and from the product term for continuous dairy amount. Benjamini-Hochberg FDR correction was applied separately to the six overall interaction tests within each outcome-modifier combination.

**Table S10. Subgroup-specific associations between dairy intake variables and depressive and anxiety symptoms stratified by region**

| Variable | Suburban |  | Urban |  | P for interaction | FDR P for interaction |
| --- | --- | --- | --- | --- | --- | --- |
|  | Beta (95%CI) | P | Beta (95%CI) | P |  |  |
| **Depressive symptoms** |  |  |  |  |  |  |
| Frequency of milk (Ref. = Never) |  |  |  |  | <0.001 | 0.001 |
| Less than once per month | -0.67 (-0.99, -0.36) | <0.001 | -0.39 (-0.70, -0.09) | 0.012 |  |  |
| 1-3 days/week | -2.04 (-2.31, -1.76) | <0.001 | -1.57 (-1.84, -1.30) | <0.001 |  |  |
| 4-6 days/week | -2.28 (-2.57, -1.99) | <0.001 | -1.58 (-1.87, -1.29) | <0.001 |  |  |
| Daily | -2.66 (-2.95, -2.38) | <0.001 | -1.94 (-2.22, -1.65) | <0.001 |  |  |
| Frequency of yogurt (Ref. = Never) |  |  |  |  | 0.629 | 0.629 |
| Less than once per month | -0.59 (-0.80, -0.37) | <0.001 | -0.91 (-1.14, -0.68) | <0.001 |  |  |
| 1-3 days/week | -0.94 (-1.15, -0.73) | <0.001 | -1.32 (-1.54, -1.10) | <0.001 |  |  |
| 4-6 days/week | -0.72 (-0.98, -0.46) | <0.001 | -0.92 (-1.19, -0.65) | <0.001 |  |  |
| Daily | -0.76 (-1.05, -0.47) | <0.001 | -1.04 (-1.34, -0.74) | <0.001 |  |  |
| Frequency of milk powder (Ref. = Never) |  |  |  |  | 0.014 | 0.020 |
| Less than once per month | 0.01 (-0.20, 0.22) | 0.919 | 0.25 (0.03, 0.48) | 0.025 |  |  |
| 1-3 days/week | 0.40 (0.17, 0.63) | <0.001 | 0.12 (-0.11, 0.36) | 0.303 |  |  |
| 4-6 days/week | 0.64 (0.28, 1.00) | <0.001 | 0.23 (-0.12, 0.59) | 0.194 |  |  |
| Daily | 0.67 (0.30, 1.04) | <0.001 | 0.71 (0.32, 1.10) | <0.001 |  |  |
| Frequency of cheese/cheese cubes (Ref. = Never) |  |  |  |  | 0.008 | 0.016 |
| Less than once per month | -0.02 (-0.27, 0.23) | 0.850 | 0.22 (-0.03, 0.47) | 0.078 |  |  |
| 1-3 days/week | 0.25 (-0.05, 0.54) | 0.101 | 0.31 (0.01, 0.61) | 0.045 |  |  |
| 4-6 days/week | 0.55 (0.11, 0.99) | 0.014 | 0.56 (0.12, 0.99) | 0.012 |  |  |
| Daily | 1.30 (0.80, 1.79) | <0.001 | 0.63 (0.13, 1.13) | 0.013 |  |  |
| Daily amount of milk, yogurt, and milk powder (200 mL units) | 0.03 (-0.06, 0.13) | 0.485 | 0.16 (0.07, 0.26) | <0.001 | 0.001 | 0.004 |
| Daily amount of cheese/cheese cubes (Ref. = Never) |  |  |  |  | 0.018 | 0.022 |
| 1-3 pieces | -0.37 (-0.61, -0.13) | 0.003 | -0.25 (-0.50, -0.01) | 0.043 |  |  |
| 4-6 pieces | 0.08 (-0.25, 0.40) | 0.641 | 0.20 (-0.13, 0.52) | 0.239 |  |  |
| 7-9 pieces | 0.80 (0.34, 1.26) | <0.001 | 1.04 (0.58, 1.51) | <0.001 |  |  |
| 10-12 pieces | 1.35 (0.77, 1.93) | <0.001 | 0.92 (0.40, 1.43) | <0.001 |  |  |
| 13-15 pieces | 2.98 (2.09, 3.87) | <0.001 | 1.43 (0.64, 2.22) | <0.001 |  |  |
| >15 pieces | 1.11 (0.27, 1.96) | 0.010 | 2.24 (1.36, 3.11) | <0.001 |  |  |
| **Anxiety symptoms** |  |  |  |  |  |  |
| Frequency of milk (Ref. = Never) |  |  |  |  | <0.001 | <0.001 |
| Less than once per month | -0.23 (-0.37, -0.08) | 0.002 | -0.17 (-0.31, -0.03) | 0.018 |  |  |
| 1-3 days/week | -0.77 (-0.89, -0.65) | <0.001 | -0.66 (-0.79, -0.54) | <0.001 |  |  |
| 4-6 days/week | -0.79 (-0.92, -0.66) | <0.001 | -0.51 (-0.64, -0.38) | <0.001 |  |  |
| Daily | -1.04 (-1.17, -0.91) | <0.001 | -0.77 (-0.90, -0.64) | <0.001 |  |  |
| Frequency of yogurt (Ref. = Never) |  |  |  |  | 0.389 | 0.389 |
| Less than once per month | 0.01 (-0.09, 0.11) | 0.881 | -0.19 (-0.30, -0.09) | <0.001 |  |  |
| 1-3 days/week | -0.30 (-0.40, -0.21) | <0.001 | -0.52 (-0.62, -0.42) | <0.001 |  |  |
| 4-6 days/week | -0.12 (-0.24, -0.00) | 0.045 | -0.36 (-0.48, -0.23) | <0.001 |  |  |
| Daily | -0.30 (-0.43, -0.17) | <0.001 | -0.42 (-0.56, -0.29) | <0.001 |  |  |
| Frequency of milk powder (Ref. = Never) |  |  |  |  | 0.220 | 0.264 |
| Less than once per month | 0.02 (-0.08, 0.11) | 0.747 | 0.02 (-0.08, 0.12) | 0.682 |  |  |
| 1-3 days/week | -0.29 (-0.39, -0.18) | <0.001 | -0.27 (-0.38, -0.16) | <0.001 |  |  |
| 4-6 days/week | -0.16 (-0.33, -0.00) | 0.048 | -0.20 (-0.36, -0.04) | 0.014 |  |  |
| Daily | -0.15 (-0.32, 0.02) | 0.084 | -0.26 (-0.44, -0.08) | 0.005 |  |  |
| Frequency of cheese/cheese cubes (Ref. = Never) |  |  |  |  | <0.001 | <0.001 |
| Less than once per month | 0.11 (-0.01, 0.22) | 0.066 | 0.13 (0.02, 0.25) | 0.025 |  |  |
| 1-3 days/week | -0.25 (-0.38, -0.11) | <0.001 | -0.10 (-0.24, 0.04) | 0.160 |  |  |
| 4-6 days/week | -0.23 (-0.43, -0.03) | 0.026 | -0.21 (-0.41, -0.01) | 0.040 |  |  |
| Daily | -0.61 (-0.83, -0.38) | <0.001 | -0.72 (-0.95, -0.49) | <0.001 |  |  |
| Daily amount of milk, yogurt, and milk powder (200 mL units) | 0.19 (0.14, 0.23) | <0.001 | 0.22 (0.17, 0.26) | <0.001 | 0.032 | 0.049 |
| Daily amount of cheese/cheese cubes (Ref. = Never) |  |  |  |  | <0.001 | <0.001 |
| 1-3 pieces | -0.15 (-0.26, -0.04) | 0.009 | 0.04 (-0.07, 0.15) | 0.476 |  |  |
| 4-6 pieces | -0.25 (-0.39, -0.10) | 0.001 | -0.01 (-0.16, 0.14) | 0.885 |  |  |
| 7-9 pieces | -0.08 (-0.29, 0.13) | 0.474 | 0.09 (-0.12, 0.30) | 0.399 |  |  |
| 10-12 pieces | 0.34 (0.08, 0.61) | 0.011 | -0.24 (-0.48, -0.01) | 0.044 |  |  |
| 13-15 pieces | 0.45 (0.04, 0.85) | 0.030 | 0.27 (-0.09, 0.63) | 0.143 |  |  |
| >15 pieces | 0.65 (0.26, 1.03) | <0.001 | 0.80 (0.40, 1.20) | <0.001 |  |  |

Note: BMI, body mass index; CI, confidence intervals; FDR, false discovery rate. Each model included all six dairy intake variables and covariates simultaneously. The P for interaction was obtained from a joint test of interaction terms for categorical dairy variables and from the product term for continuous dairy amount. Benjamini-Hochberg FDR correction was applied separately to the six overall interaction tests within each outcome-modifier combination.

**Table S11. Sensitivity analyses of the associations between dairy intake and depressive and anxiety symptoms using school-level random-intercept models**

| Variable | Depressive symptoms | | Anxiety symptoms | |
| --- | --- | --- | --- | --- |
|  | beta (95% CI) | P | beta (95% CI) | P |
| Frequency of milk (Ref. = Never) |  |  |  |  |
| Less than once per month | -0.48 (-0.69, -0.26) | <0.001 | -0.19 (-0.28, -0.09) | <0.001 |
| 1-3 days/week | -1.67 (-1.86, -1.48) | <0.001 | -0.64 (-0.73, -0.56) | <0.001 |
| 4-6 days/week | -1.78 (-1.98, -1.58) | <0.001 | -0.62 (-0.71, -0.53) | <0.001 |
| Daily | -2.16 (-2.36, -1.96) | <0.001 | -0.85 (-0.94, -0.76) | <0.001 |
| Frequency of yogurt (Ref. = Never) |  |  |  |  |
| Less than once per month | -0.69 (-0.84, -0.53) | <0.001 | -0.06 (-0.14, 0.01) | 0.071 |
| 1-3 days/week | -1.02 (-1.17, -0.87) | <0.001 | -0.33 (-0.40, -0.27) | <0.001 |
| 4-6 days/week | -0.76 (-0.94, -0.57) | <0.001 | -0.23 (-0.32, -0.15) | <0.001 |
| Daily | -0.73 (-0.94, -0.52) | <0.001 | -0.29 (-0.38, -0.19) | <0.001 |
| Frequency of milk powder (Ref. = Never) |  |  |  |  |
| Less than once per month | 0.14 (-0.01, 0.29) | 0.073 | 0.06 (-0.01, 0.13) | 0.109 |
| 1-3 days/week | 0.41 (0.25, 0.58) | <0.001 | -0.15 (-0.23, -0.08) | <0.001 |
| 4-6 days/week | 0.75 (0.50, 1.00) | <0.001 | 0.01 (-0.10, 0.12) | 0.849 |
| Daily | 0.72 (0.45, 0.99) | <0.001 | -0.11 (-0.23, 0.01) | 0.082 |
| Frequency of cheese/cheese cubes (Ref. = Never) |  |  |  |  |
| Less than once per month | 0.07 (-0.10, 0.25) | 0.406 | 0.11 (0.03, 0.19) | 0.006 |
| 1-3 days/week | 0.32 (0.11, 0.53) | 0.003 | -0.09 (-0.19, 0.00) | 0.063 |
| 4-6 days/week | 0.60 (0.29, 0.90) | <0.001 | -0.12 (-0.26, 0.02) | 0.096 |
| Daily | 1.09 (0.74, 1.43) | <0.001 | -0.51 (-0.67, -0.35) | <0.001 |
| Daily amount of milk, yogurt, and milk powder (200 mL units) | -0.01 (-0.08, 0.05) | 0.673 | 0.13 (0.10, 0.16) | <0.001 |
| Daily amount of cheese/cheese cubes (Ref. = Never) |  |  |  |  |
| 1-3 pieces | -0.37 (-0.54, -0.19) | <0.001 | -0.10 (-0.18, -0.02) | 0.013 |
| 4-6 pieces | 0.04 (-0.19, 0.26) | 0.748 | -0.20 (-0.30, -0.10) | <0.001 |
| 7-9 pieces | 0.85 (0.53, 1.18) | <0.001 | 0.02 (-0.13, 0.16) | 0.841 |
| 10-12 pieces | 1.40 (1.02, 1.79) | <0.001 | 0.16 (-0.01, 0.34) | 0.065 |
| 13-15 pieces | 2.26 (1.68, 2.85) | <0.001 | 0.45 (0.19, 0.72) | <0.001 |
| >15 pieces | 1.49 (0.89, 2.09) | <0.001 | 0.66 (0.39, 0.93) | <0.001 |

Note: BMI, body mass index; CI, confidence intervals.

**Table S12. School-level mixed-effects interaction analysis of dairy intake with gender for depressive and anxiety symptoms**

| Outcome | Model | Modifier | Dairy intake | Interaction term | Beta (95%CI) | P value | P for interaction | FDR P for interaction |
| --- | --- | --- | --- | --- | --- | --- | --- | --- |
| Depressive symptoms | Model 1 | Gender (Ref. = Girls) | Frequency of milk (Ref. = Never) | Less than once per month × Boys | -0.11 (-0.53, 0.30) | 0.598 | <0.001 | <0.001 |
| Depressive symptoms | Model 1 | Gender (Ref. = Girls) | Frequency of milk (Ref. = Never) | 1-3 days/week × Boys | 0.54 (0.18, 0.89) | 0.003 |  |  |
| Depressive symptoms | Model 1 | Gender (Ref. = Girls) | Frequency of milk (Ref. = Never) | 4-6 days/week × Boys | 0.62 (0.24, 1.00) | 0.001 |  |  |
| Depressive symptoms | Model 1 | Gender (Ref. = Girls) | Frequency of milk (Ref. = Never) | Daily × Boys | 0.35 (-0.02, 0.71) | 0.063 |  |  |
| Depressive symptoms | Model 2 | Gender (Ref. = Girls) | Frequency of yogurt (Ref. = Never) | Less than once per month × Boys | 0.43 (0.13, 0.73) | 0.005 | <0.001 | <0.001 |
| Depressive symptoms | Model 2 | Gender (Ref. = Girls) | Frequency of yogurt (Ref. = Never) | 1-3 days/week × Boys | 0.89 (0.61, 1.17) | <0.001 |  |  |
| Depressive symptoms | Model 2 | Gender (Ref. = Girls) | Frequency of yogurt (Ref. = Never) | 4-6 days/week × Boys | 0.59 (0.25, 0.93) | <0.001 |  |  |
| Depressive symptoms | Model 2 | Gender (Ref. = Girls) | Frequency of yogurt (Ref. = Never) | Daily × Boys | 0.72 (0.36, 1.08) | <0.001 |  |  |
| Depressive symptoms | Model 3 | Gender (Ref. = Girls) | Frequency of milk powder (Ref. = Never) | Less than once per month × Boys | 0.62 (0.33, 0.91) | <0.001 | <0.001 | <0.001 |
| Depressive symptoms | Model 3 | Gender (Ref. = Girls) | Frequency of milk powder (Ref. = Never) | 1-3 days/week × Boys | 0.92 (0.64, 1.20) | <0.001 |  |  |
| Depressive symptoms | Model 3 | Gender (Ref. = Girls) | Frequency of milk powder (Ref. = Never) | 4-6 days/week × Boys | 1.34 (0.92, 1.77) | <0.001 |  |  |
| Depressive symptoms | Model 3 | Gender (Ref. = Girls) | Frequency of milk powder (Ref. = Never) | Daily × Boys | 0.69 (0.29, 1.09) | <0.001 |  |  |
| Depressive symptoms | Model 4 | Gender (Ref. = Girls) | Frequency of cheese/cheese cubes (Ref. = Never) | Less than once per month × Boys | 0.11 (-0.13, 0.35) | 0.372 | <0.001 | <0.001 |
| Depressive symptoms | Model 4 | Gender (Ref. = Girls) | Frequency of cheese/cheese cubes (Ref. = Never) | 1-3 days/week × Boys | 0.81 (0.54, 1.09) | <0.001 |  |  |
| Depressive symptoms | Model 4 | Gender (Ref. = Girls) | Frequency of cheese/cheese cubes (Ref. = Never) | 4-6 days/week × Boys | 1.23 (0.76, 1.69) | <0.001 |  |  |
| Depressive symptoms | Model 4 | Gender (Ref. = Girls) | Frequency of cheese/cheese cubes (Ref. = Never) | Daily × Boys | 0.84 (0.37, 1.31) | <0.001 |  |  |
| Depressive symptoms | Model 5 | Gender (Ref. = Girls) | Daily amount of milk, yogurt, and milk powder (200 mL units) | Daily amount of milk, yogurt, and milk powder (200 mL units) × Boys | -0.21 (-0.33, -0.08) | 0.001 | 0.001 | 0.001 |
| Depressive symptoms | Model 6 | Gender (Ref. = Girls) | Daily amount of cheese/cheese cubes (Ref. = Never) | 1-3 pieces × Boys | 0.35 (0.14, 0.57) | 0.001 | <0.001 | <0.001 |
| Depressive symptoms | Model 6 | Gender (Ref. = Girls) | Daily amount of cheese/cheese cubes (Ref. = Never) | 4-6 pieces × Boys | 0.34 (-0.00, 0.68) | 0.051 |  |  |
| Depressive symptoms | Model 6 | Gender (Ref. = Girls) | Daily amount of cheese/cheese cubes (Ref. = Never) | 7-9 pieces × Boys | 0.96 (0.40, 1.52) | <0.001 |  |  |
| Depressive symptoms | Model 6 | Gender (Ref. = Girls) | Daily amount of cheese/cheese cubes (Ref. = Never) | 10-12 pieces × Boys | 1.68 (1.01, 2.35) | <0.001 |  |  |
| Depressive symptoms | Model 6 | Gender (Ref. = Girls) | Daily amount of cheese/cheese cubes (Ref. = Never) | 13-15 pieces × Boys | 1.83 (0.64, 3.02) | 0.003 |  |  |
| Depressive symptoms | Model 6 | Gender (Ref. = Girls) | Daily amount of cheese/cheese cubes (Ref. = Never) | >15 pieces × Boys | -0.62 (-1.83, 0.58) | 0.312 |  |  |
| Depressive symptoms | Model 7 | Region (Ref. = Suburban) | Frequency of milk (Ref. = Never) | Less than once per month × Urban | 0.46 (0.04, 0.87) | 0.030 | <0.001 | 0.005 |
| Depressive symptoms | Model 7 | Region (Ref. = Suburban) | Frequency of milk (Ref. = Never) | 1-3 days/week × Urban | 0.53 (0.18, 0.89) | 0.003 |  |  |
| Depressive symptoms | Model 7 | Region (Ref. = Suburban) | Frequency of milk (Ref. = Never) | 4-6 days/week × Urban | 0.75 (0.37, 1.12) | <0.001 |  |  |
| Depressive symptoms | Model 7 | Region (Ref. = Suburban) | Frequency of milk (Ref. = Never) | Daily × Urban | 0.71 (0.35, 1.08) | <0.001 |  |  |
| Depressive symptoms | Model 8 | Region (Ref. = Suburban) | Frequency of yogurt (Ref. = Never) | Less than once per month × Urban | -0.02 (-0.32, 0.27) | 0.870 | 0.835 | 0.835 |
| Depressive symptoms | Model 8 | Region (Ref. = Suburban) | Frequency of yogurt (Ref. = Never) | 1-3 days/week × Urban | -0.10 (-0.38, 0.17) | 0.467 |  |  |
| Depressive symptoms | Model 8 | Region (Ref. = Suburban) | Frequency of yogurt (Ref. = Never) | 4-6 days/week × Urban | 0.02 (-0.31, 0.36) | 0.886 |  |  |
| Depressive symptoms | Model 8 | Region (Ref. = Suburban) | Frequency of yogurt (Ref. = Never) | Daily × Urban | 0.03 (-0.33, 0.39) | 0.857 |  |  |
| Depressive symptoms | Model 9 | Region (Ref. = Suburban) | Frequency of milk powder (Ref. = Never) | Less than once per month × Urban | 0.42 (0.13, 0.71) | 0.005 | 0.058 | 0.087 |
| Depressive symptoms | Model 9 | Region (Ref. = Suburban) | Frequency of milk powder (Ref. = Never) | 1-3 days/week × Urban | -0.09 (-0.38, 0.20) | 0.540 |  |  |
| Depressive symptoms | Model 9 | Region (Ref. = Suburban) | Frequency of milk powder (Ref. = Never) | 4-6 days/week × Urban | -0.02 (-0.46, 0.41) | 0.915 |  |  |
| Depressive symptoms | Model 9 | Region (Ref. = Suburban) | Frequency of milk powder (Ref. = Never) | Daily × Urban | -0.04 (-0.44, 0.36) | 0.832 |  |  |
| Depressive symptoms | Model 10 | Region (Ref. = Suburban) | Frequency of cheese/cheese cubes (Ref. = Never) | Less than once per month × Urban | 0.34 (0.10, 0.58) | 0.005 | 0.050 | 0.087 |
| Depressive symptoms | Model 10 | Region (Ref. = Suburban) | Frequency of cheese/cheese cubes (Ref. = Never) | 1-3 days/week × Urban | 0.03 (-0.25, 0.31) | 0.839 |  |  |
| Depressive symptoms | Model 10 | Region (Ref. = Suburban) | Frequency of cheese/cheese cubes (Ref. = Never) | 4-6 days/week × Urban | 0.22 (-0.24, 0.69) | 0.348 |  |  |
| Depressive symptoms | Model 10 | Region (Ref. = Suburban) | Frequency of cheese/cheese cubes (Ref. = Never) | Daily × Urban | -0.17 (-0.64, 0.29) | 0.465 |  |  |
| Depressive symptoms | Model 11 | Region (Ref. = Suburban) | Daily amount of milk, yogurt, and milk powder (200 mL units) | Daily amount of milk, yogurt, and milk powder (200 mL units) × Urban | 0.17 (0.05, 0.30) | 0.007 | 0.007 | 0.020 |
| Depressive symptoms | Model 12 | Region (Ref. = Suburban) | Daily amount of cheese/cheese cubes (Ref. = Never) | 1-3 pieces × Urban | 0.21 (-0.01, 0.43) | 0.059 | 0.107 | 0.128 |
| Depressive symptoms | Model 12 | Region (Ref. = Suburban) | Daily amount of cheese/cheese cubes (Ref. = Never) | 4-6 pieces × Urban | 0.20 (-0.15, 0.55) | 0.270 |  |  |
| Depressive symptoms | Model 12 | Region (Ref. = Suburban) | Daily amount of cheese/cheese cubes (Ref. = Never) | 7-9 pieces × Urban | 0.32 (-0.25, 0.88) | 0.273 |  |  |
| Depressive symptoms | Model 12 | Region (Ref. = Suburban) | Daily amount of cheese/cheese cubes (Ref. = Never) | 10-12 pieces × Urban | 0.37 (-0.32, 1.06) | 0.290 |  |  |
| Depressive symptoms | Model 12 | Region (Ref. = Suburban) | Daily amount of cheese/cheese cubes (Ref. = Never) | 13-15 pieces × Urban | -0.98 (-2.11, 0.15) | 0.088 |  |  |
| Depressive symptoms | Model 12 | Region (Ref. = Suburban) | Daily amount of cheese/cheese cubes (Ref. = Never) | >15 pieces × Urban | 0.77 (-0.38, 1.92) | 0.192 |  |  |
| Anxiety symptoms | Model 13 | Gender (Ref. = Girls) | Frequency of milk (Ref. = Never) | Less than once per month × Boys | 0.04 (-0.15, 0.23) | 0.688 | <0.001 | <0.001 |
| Anxiety symptoms | Model 13 | Gender (Ref. = Girls) | Frequency of milk (Ref. = Never) | 1-3 days/week × Boys | 0.26 (0.10, 0.42) | 0.001 |  |  |
| Anxiety symptoms | Model 13 | Gender (Ref. = Girls) | Frequency of milk (Ref. = Never) | 4-6 days/week × Boys | 0.33 (0.15, 0.50) | <0.001 |  |  |
| Anxiety symptoms | Model 13 | Gender (Ref. = Girls) | Frequency of milk (Ref. = Never) | Daily × Boys | 0.24 (0.07, 0.40) | 0.005 |  |  |
| Anxiety symptoms | Model 14 | Gender (Ref. = Girls) | Frequency of yogurt (Ref. = Never) | Less than once per month × Boys | 0.16 (0.02, 0.30) | 0.022 | <0.001 | <0.001 |
| Anxiety symptoms | Model 14 | Gender (Ref. = Girls) | Frequency of yogurt (Ref. = Never) | 1-3 days/week × Boys | 0.32 (0.19, 0.45) | <0.001 |  |  |
| Anxiety symptoms | Model 14 | Gender (Ref. = Girls) | Frequency of yogurt (Ref. = Never) | 4-6 days/week × Boys | 0.14 (-0.02, 0.29) | 0.081 |  |  |
| Anxiety symptoms | Model 14 | Gender (Ref. = Girls) | Frequency of yogurt (Ref. = Never) | Daily × Boys | 0.11 (-0.05, 0.28) | 0.183 |  |  |
| Anxiety symptoms | Model 15 | Gender (Ref. = Girls) | Frequency of milk powder (Ref. = Never) | Less than once per month × Boys | 0.34 (0.21, 0.47) | <0.001 | <0.001 | <0.001 |
| Anxiety symptoms | Model 15 | Gender (Ref. = Girls) | Frequency of milk powder (Ref. = Never) | 1-3 days/week × Boys | 0.47 (0.34, 0.59) | <0.001 |  |  |
| Anxiety symptoms | Model 15 | Gender (Ref. = Girls) | Frequency of milk powder (Ref. = Never) | 4-6 days/week × Boys | 0.55 (0.35, 0.74) | <0.001 |  |  |
| Anxiety symptoms | Model 15 | Gender (Ref. = Girls) | Frequency of milk powder (Ref. = Never) | Daily × Boys | 0.29 (0.11, 0.47) | 0.002 |  |  |
| Anxiety symptoms | Model 16 | Gender (Ref. = Girls) | Frequency of cheese/cheese cubes (Ref. = Never) | Less than once per month × Boys | 0.06 (-0.05, 0.16) | 0.304 | <0.001 | <0.001 |
| Anxiety symptoms | Model 16 | Gender (Ref. = Girls) | Frequency of cheese/cheese cubes (Ref. = Never) | 1-3 days/week × Boys | 0.34 (0.21, 0.46) | <0.001 |  |  |
| Anxiety symptoms | Model 16 | Gender (Ref. = Girls) | Frequency of cheese/cheese cubes (Ref. = Never) | 4-6 days/week × Boys | 0.42 (0.21, 0.63) | <0.001 |  |  |
| Anxiety symptoms | Model 16 | Gender (Ref. = Girls) | Frequency of cheese/cheese cubes (Ref. = Never) | Daily × Boys | 0.28 (0.07, 0.49) | 0.011 |  |  |
| Anxiety symptoms | Model 17 | Gender (Ref. = Girls) | Daily amount of milk, yogurt, and milk powder (200 mL units) | Daily amount of milk, yogurt, and milk powder (200 mL units) × Boys | -0.05 (-0.11, 0.00) | 0.069 | 0.069 | 0.069 |
| Anxiety symptoms | Model 18 | Gender (Ref. = Girls) | Daily amount of cheese/cheese cubes (Ref. = Never) | 1-3 pieces × Boys | 0.10 (0.01, 0.20) | 0.038 | <0.001 | <0.001 |
| Anxiety symptoms | Model 18 | Gender (Ref. = Girls) | Daily amount of cheese/cheese cubes (Ref. = Never) | 4-6 pieces × Boys | 0.14 (-0.02, 0.29) | 0.079 |  |  |
| Anxiety symptoms | Model 18 | Gender (Ref. = Girls) | Daily amount of cheese/cheese cubes (Ref. = Never) | 7-9 pieces × Boys | 0.21 (-0.05, 0.46) | 0.112 |  |  |
| Anxiety symptoms | Model 18 | Gender (Ref. = Girls) | Daily amount of cheese/cheese cubes (Ref. = Never) | 10-12 pieces × Boys | 0.75 (0.44, 1.05) | <0.001 |  |  |
| Anxiety symptoms | Model 18 | Gender (Ref. = Girls) | Daily amount of cheese/cheese cubes (Ref. = Never) | 13-15 pieces × Boys | 0.30 (-0.24, 0.84) | 0.274 |  |  |
| Anxiety symptoms | Model 18 | Gender (Ref. = Girls) | Daily amount of cheese/cheese cubes (Ref. = Never) | >15 pieces × Boys | -0.66 (-1.20, -0.11) | 0.019 |  |  |
| Anxiety symptoms | Model 19 | Region (Ref. = Suburban) | Frequency of milk (Ref. = Never) | Less than once per month × Urban | 0.11 (-0.08, 0.30) | 0.245 | 0.033 | 0.067 |
| Anxiety symptoms | Model 19 | Region (Ref. = Suburban) | Frequency of milk (Ref. = Never) | 1-3 days/week × Urban | 0.11 (-0.06, 0.27) | 0.198 |  |  |
| Anxiety symptoms | Model 19 | Region (Ref. = Suburban) | Frequency of milk (Ref. = Never) | 4-6 days/week × Urban | 0.21 (0.04, 0.38) | 0.016 |  |  |
| Anxiety symptoms | Model 19 | Region (Ref. = Suburban) | Frequency of milk (Ref. = Never) | Daily × Urban | 0.21 (0.05, 0.38) | 0.011 |  |  |
| Anxiety symptoms | Model 20 | Region (Ref. = Suburban) | Frequency of yogurt (Ref. = Never) | Less than once per month × Urban | -0.09 (-0.22, 0.05) | 0.195 | 0.371 | 0.371 |
| Anxiety symptoms | Model 20 | Region (Ref. = Suburban) | Frequency of yogurt (Ref. = Never) | 1-3 days/week × Urban | -0.07 (-0.20, 0.05) | 0.267 |  |  |
| Anxiety symptoms | Model 20 | Region (Ref. = Suburban) | Frequency of yogurt (Ref. = Never) | 4-6 days/week × Urban | -0.13 (-0.28, 0.02) | 0.099 |  |  |
| Anxiety symptoms | Model 20 | Region (Ref. = Suburban) | Frequency of yogurt (Ref. = Never) | Daily × Urban | 0.00 (-0.16, 0.16) | 0.983 |  |  |
| Anxiety symptoms | Model 21 | Region (Ref. = Suburban) | Frequency of milk powder (Ref. = Never) | Less than once per month × Urban | 0.10 (-0.03, 0.23) | 0.133 | 0.370 | 0.371 |
| Anxiety symptoms | Model 21 | Region (Ref. = Suburban) | Frequency of milk powder (Ref. = Never) | 1-3 days/week × Urban | 0.10 (-0.03, 0.23) | 0.126 |  |  |
| Anxiety symptoms | Model 21 | Region (Ref. = Suburban) | Frequency of milk powder (Ref. = Never) | 4-6 days/week × Urban | 0.03 (-0.17, 0.23) | 0.786 |  |  |
| Anxiety symptoms | Model 21 | Region (Ref. = Suburban) | Frequency of milk powder (Ref. = Never) | Daily × Urban | -0.02 (-0.20, 0.16) | 0.857 |  |  |
| Anxiety symptoms | Model 22 | Region (Ref. = Suburban) | Frequency of cheese/cheese cubes (Ref. = Never) | Less than once per month × Urban | 0.17 (0.07, 0.28) | 0.002 | 0.001 | 0.004 |
| Anxiety symptoms | Model 22 | Region (Ref. = Suburban) | Frequency of cheese/cheese cubes (Ref. = Never) | 1-3 days/week × Urban | 0.21 (0.08, 0.34) | 0.001 |  |  |
| Anxiety symptoms | Model 22 | Region (Ref. = Suburban) | Frequency of cheese/cheese cubes (Ref. = Never) | 4-6 days/week × Urban | 0.07 (-0.15, 0.28) | 0.536 |  |  |
| Anxiety symptoms | Model 22 | Region (Ref. = Suburban) | Frequency of cheese/cheese cubes (Ref. = Never) | Daily × Urban | -0.01 (-0.22, 0.20) | 0.945 |  |  |
| Anxiety symptoms | Model 23 | Region (Ref. = Suburban) | Daily amount of milk, yogurt, and milk powder (200 mL units) | Daily amount of milk, yogurt, and milk powder (200 mL units) × Urban | 0.05 (-0.00, 0.11) | 0.071 | 0.071 | 0.107 |
| Anxiety symptoms | Model 24 | Region (Ref. = Suburban) | Daily amount of cheese/cheese cubes (Ref. = Never) | 1-3 pieces × Urban | 0.23 (0.13, 0.33) | <0.001 | <0.001 | <0.001 |
| Anxiety symptoms | Model 24 | Region (Ref. = Suburban) | Daily amount of cheese/cheese cubes (Ref. = Never) | 4-6 pieces × Urban | 0.21 (0.05, 0.37) | 0.010 |  |  |
| Anxiety symptoms | Model 24 | Region (Ref. = Suburban) | Daily amount of cheese/cheese cubes (Ref. = Never) | 7-9 pieces × Urban | 0.27 (0.01, 0.53) | 0.039 |  |  |
| Anxiety symptoms | Model 24 | Region (Ref. = Suburban) | Daily amount of cheese/cheese cubes (Ref. = Never) | 10-12 pieces × Urban | -0.34 (-0.65, -0.03) | 0.033 |  |  |
| Anxiety symptoms | Model 24 | Region (Ref. = Suburban) | Daily amount of cheese/cheese cubes (Ref. = Never) | 13-15 pieces × Urban | -0.01 (-0.52, 0.50) | 0.969 |  |  |
| Anxiety symptoms | Model 24 | Region (Ref. = Suburban) | Daily amount of cheese/cheese cubes (Ref. = Never) | >15 pieces × Urban | 0.03 (-0.49, 0.55) | 0.917 |  |  |

Note: BMI, body mass index; CI, confidence intervals; FDR, false discovery rate. Each model included all six dairy intake variables and covariates simultaneously, with one interaction term added at a time. The P for interaction was obtained from a joint test of interaction terms for categorical dairy variables and from the product term for continuous dairy amount. Benjamini-Hochberg FDR correction was applied separately to the six overall interaction tests within each outcome-modifier combination.

**Figure S1. Directed acyclic graph of the hypothesized relationships between dairy intake and depressive and anxiety symptoms**

Note: Arrows indicate hypothesized causal directions. Sleep duration and weight dissatisfaction were specified as potential mediators, whereas the remaining variables were considered potential confounders. The DAG was used to guide covariate selection for the multivariable analyses.

**Figure S2. Restricted cubic spline associations between dairy intake variables and depressive symptoms in the primary mutually adjusted models**

Note: All six dairy intake variables were included simultaneously. Models were adjusted for age, gender, ethnicity, education level, region, body mass index, living on campus, history of smoking, history of drinking, physical inactivity, low fruit or vegetable intake, unhealthy diet, father’s education level, and mother’s education level. Shaded areas represent 95% confidence intervals.

**Figure S3. Restricted cubic spline associations between dairy intake variables and anxiety symptoms in the primary mutually adjusted models**

Note: All six dairy intake variables were included simultaneously. Models were adjusted for age, gender, ethnicity, education level, region, body mass index, living on campus, history of smoking, history of drinking, physical inactivity, low fruit or vegetable intake, unhealthy diet, father’s education level, and mother’s education level. Shaded areas represent 95% confidence intervals.

**Figure S4. Restricted cubic spline associations between dairy intake variables and depressive symptoms in the single-exposure covariate-adjusted models**

Note: Each dairy intake variable was entered separately. Models were adjusted for age, gender, ethnicity, education level, region, body mass index, living on campus, history of smoking, history of drinking, physical inactivity, low fruit or vegetable intake, unhealthy diet, father’s education level, and mother’s education level. Shaded areas represent 95% confidence intervals.

**Figure S5. Restricted cubic spline associations between dairy intake variables and anxiety symptoms in the single-exposure covariate-adjusted models**

Note: Each dairy intake variable was entered separately. Models were adjusted for age, gender, ethnicity, education level, region, body mass index, living on campus, history of smoking, history of drinking, physical inactivity, low fruit or vegetable intake, unhealthy diet, father’s education level, and mother’s education level. Shaded areas represent 95% confidence intervals.
